# Revision of the Afrotropical endemic *Eumerus triangularis* group (Diptera: Syrphidae: Merodontini) – Species with glistering antennae

**DOI:** 10.1371/journal.pone.0313829

**Published:** 2025-03-24

**Authors:** Snežana Radenković, Nevena Veličković, Kurt Jordaens, Ana S. Grković, Mihajla Djan, Gunilla Ståhls, John Smit, Ante Vujić

**Affiliations:** 1 Faculty of Sciences, University of Novi Sad, Novi Sad, Serbia; 2 Department of Biology, Entomology Section, Royal Museum for Central Africa, Tervuren, Belgium; 3 Finnish Museum of Natural History, Zoology Unit, University of Helsinki, Helsinki, Finland; 4 European Invertebrate Survey – the Netherlands, Leiden, the Netherlands; The American College, INDIA

## Abstract

The *Eumerus triangularis* group (Diptera: Syrphidae: Merodontini), which is endemic to the Afrotropical Region, has been thoroughly reviewed. This study identifies nine species within the group, with four of them redescribed: *E*. *rubidus* Hull, 1964, *E*. *tessellatus* Hull, 1964, *E*. *triangularis* Hervé-Bazin, 1913, and *E*. *villeneuvei* Hervé-Bazin, 1913 and with the description of four new species – *E*. *argentipedicellus* Radenković, Vujić & Grković sp. nov., *E*. *brunnipennis* Radenković, Vujić & Grković sp. nov., *E*. *clavicercus* Radenković, Vujić & Grković sp. nov., and *E*. *setifemoratus* Radenković, Vujić & Grković sp. nov. Furthermore, the previously unknown male of *E*. *rubidus* and the female of *E*. *tessellatus* are described. This study includes illustrated identification keys for males and females, along with detailed drawings of male terminalia for all nine species. DNA sequences of two regions of the mitochondrial DNA, cytochrome c oxidase subunit 1 (COI) gene and cytochrome b, and a region of the nuclear ribosomal 28rRNA gene are used to confirm morphological identification. Both molecular and morphological analyses indicate the presence of two distinct clades within the *E*. *triangularis* species group.

## Introduction

The Afrotropical Region is home to around 600 species of hoverflies (Diptera, Syrphidae) across 73 genera, currently classified into three subfamilies: Eristalinae, Microdontinae, and Syrphinae. Pipizinae is the only subfamily not found in the region. However, recent molecular studies suggest that the classification of subfamily Eristalinae which include some of the most species-rich genera in the region, such as *Eumerus* Meigen, 1804, *Eristalinus* Rondani, 1845, and *Ceriana* Rafinesque, 1815 [1], may need revision, with some tribes, such as Merodontini and Volucellini, potentially being elevated to separate subfamilies [2–4]. Despite expeditions of the Old World started at the beginning of 20^th^ century, published papers on hoverflies are relatively rare and often restricted to specific areas or particular taxa [5–37].

Identifying species within certain larger genera remains difficult due to incomplete identification keys, unclear taxonomy, and the presence of many species waiting to be described [1,2]. To aid in identifying species and discovering potential new species, Jordaens et al. [25] used DNA barcoding to create a reference database of 523 DNA barcodes for 98 Afrotropical hoverfly species. Their results showed that the DNA reference database is still largely incomplete, and species from several genera could not be morphologically identified to the species level due to the lack of identification keys and the aforementioned issues, limiting the database’s usefulness for species identification in these genera.

A case in point is the genus *Eumerus* Meigen, 1822 which is widely distributed in the Palaearctic, Afrotropical, Oriental and Australasian Regions, with ca. 300 described species, of which more than 77 occur in the Afrotropical Region [2,34,38]. It is therefore one of the most (if not the most) speciose hoverfly genus in the Afrotropical Region. Some *Eumerus* species have been introduced into the Australasian, Nearctic and Neotropical Regions [2,38]. *Eumerus* are small- to medium-sized, flat-faced compact flies, often with white to greyish pruinose fasciae on the otherwise dark or reddish abdomen. Species of this genus have a characteristic wing venation with a recurrent M1 vein and lacking hairs on the lower calypter [23]. The latter character distinguishes the genus from the related endemic Afrotropical genus *Megatrigon* Johnson, 1898 [23]. *Eumerus* hoverflies are also characterised by the lateral fossette of postpedicel (usually dorsal in *Merodon*), a more or less thickened hind femur with two rows of strong setae apicoventrally and the male genitalia with an L-shaped hamus of the hypandrium [17].

Only limited taxonomic work has been conducted on the genus *Eumerus* of the Afrotropical Region. Bezzi [5] was the first to provide an identification key for males of 11 *Eumerus* species in the (western) Afrotropical Region, one of which is currently placed in *Megatrigon*. Hervé-Bazin [38] complemented Bezzi’s [5] key with another 7 *Eumerus* species, including one that is now placed in *Megatrigon*. Curran [7], as *Citibaena* Walker, 1856 (misspelled as “*Citabaena*”), published an identification key for 30 African *Eumerus* species (containing two *Megatrigon* species) and described five new species, of which *E. efflatouni* Curran, 1938 has been synonymised with *E. vestitus* Bezzi, 1912 [24]. Hull [11] studied the Diptera collection of the South African expedition (1950–1951) by the University of Lund (Sweden). He described 17 new species of *Eumerus*, including four species which are currently included in *Megatrigon* {and one of which, *E. connexus* Hull, 1964 has been synonymised with *M*. *jacobi* (Hervé-Bazin, 1913) [23]}. Four of the new *Eumerus* species were described based on females only and were not considered in his identification key which was for males only, and which comprised 31 *Eumerus* and 4 *Megatrigon* species.

Leif Lyneborg (1932–2006), a former curator emeritus at the Department of Entomology at the Natural History Museum of Denmark, left an unpublished identification key for Afrotropical *Eumerus* species, with more than 75 species new to science (among others 9 new species of the genus *Megatrigon*) and 68 described *Eumerus* species included. Unfortunately, his study never got published, species descriptions are short and lack sufficient details. Descriptions of the male terminalia, which show relevant taxonomic characters in the genus, were not included.

Smit *et al.* [24] published a checklist of the hoverflies of the Arabian Peninsula, described three new *Eumerus* species, and provided an identification key to the 12 *Eumerus* species of the Arabian Peninsula. Based on the synapomorphic characters (see further: diagnosis of the *E. triangularis* group), Smit *et al.* [24] putatively delineated a group of species related to *E. triangularis* Hervé-Bazin, 1913, to which *E. lacertosus* Smit, 2017 and *E. lunatus* (Fabricius, 1794) would also belong, however, without studying the type material of the latter.

The poor taxonomy of the genus is unfortunate since the genus appears to be remarkably speciose in the Afrotropical Region and species occur in a variety of habitat types where they are associated with the plant species in which their larvae develop. *Eumerus* species occur in open, semi-humid to dry grasslands, open forests and savannas, karoo vegetation and semi-deserts, with *Aloe* (Asphodeloideae) species and rocky outcrops with sparse vegetation but also in urban gardens, parks and nurseries, where some species are regarded as pests causing damage to a variety of bulb species [2]. However, a better understanding of the co-evolution between *Eumerus* species and their host plants, the ecology of *Eumerus* and the speciation patterns in the genus in the Afrotropical Region requires an improved taxonomy and phylogeny of the genus. The limited taxonomic understanding of the genus is also reflected in the current DNA barcode reference dataset of Jordaens *et al.* [25] where only 50 DNA barcodes of eight *Eumerus* morphospecies were included, of which specimens of only four species were identified using existing identification keys and species descriptions.

This paper conducts a comprehensive examination of the *Eumerus triangularis* species group, which is endemic to the Afrotropical Region. It provides an enhanced species-group diagnosis (refer to Smit *et al*., [24]), (re)descriptions for all presently known species (except for the recently described *E. lacertosus*), an identification key for both males and females, and a distribution map of the species.

Since there is a quite evident sexual dimorphism in some of the species making it difficult to associate the two sexes [7] and existing identification keys for Afrotropical *Eumerus* are for males only [5,7,11,39], we also conducted molecular genetic analyses to link females to males. In addition, we examined relationships among the species within the species-group, based on molecular data.

## Materials and methods

### Examined collections

Specimens from the following institutional and private collections were studied:

BMSA – Bloemfontein Museum of South Africa, Bloemfontein, South Africa;
CSCA – California State Collection of Arthropods, Sacramento, USA;
FSUNS – Faculty of Sciences, Department of Biology and Ecology, University of Novi Sad, Novi Sad, Serbia;
KMMA – Koninklijk Museum voor Midden Afrika (RMCA – Royal Museum for Central Africa), Tervuren, Belgium;
MBPC – Miroslav Bartak’s Personal Collection, Czech Republic;
MZH – Finnish Museum of Natural History, University of Helsinki, Helsinki, Finland;
MZLU – Lund Museum of Zoology, Lund, Sweden;
NMSA – KwaZulu-Natal Museum, Pietermaritzburg, South Africa;
RMNH – Naturalis Biodiversity Centre, Leiden, the Netherlands;
SAMC – Iziko Museum of South Africa (formerly South African Museum), Cape Town, South Africa;
ZFMK – Zoologisches Forschungsmuseum Alexander Koenig, Bonn, Germany
ZMUC – Zoological Museum, University of Copenhagen, Copenhagen, Denmark

### Morphological analysis

The morphological terminology used in the descriptions and drawings followed Cumming and Wood [40], except for the “notopleural suture” and the terminology of the male terminalia which followed Doczkal [41].

Morphological characters were observed using a Nikon SMZ 745T stereomicroscope (Nikon, Tokyo, Japan). To study the male terminalia, dry specimens were relaxed and the terminalia were separated from the rest of the specimen using an insect pin. Terminalia were cleared by boiling in a KOH solution for 3 min. This was followed by brief immersion in acetic acid to neutralise the KOH, followed by immersion in ethanol to remove the acid. Terminalia were kept in glycerol in microvials and added to the specimen pins. A Leica MZ16 binocular microscope (Leica Microsystems, Wetzlar, Germany) with an FSA 25 PE drawing tube and a Leica DFC 320 digital camera (Leica Microsystems, Wetzlar, Germany) were used for drawings and photographs, respectively. Photographs were stacked using CombineZ software v. 5 (http://www.hadleyweb.pwp.blueyonder.co.uk/CZ5/combinez5.htm). Measurements were taken with an eyepiece graticule or micrometer and are expressed in mm.

Body length was measured in lateral view from the lunula of the head to the end of the abdomen. Wing length was measured from the tegula to the apex of the wing. Antennal length was measured from the inner side (as shown in Fig 4). The width of the female frons relative to the width of the head was measured in anterior view, in the line above the antennae.

**Fig 1:**
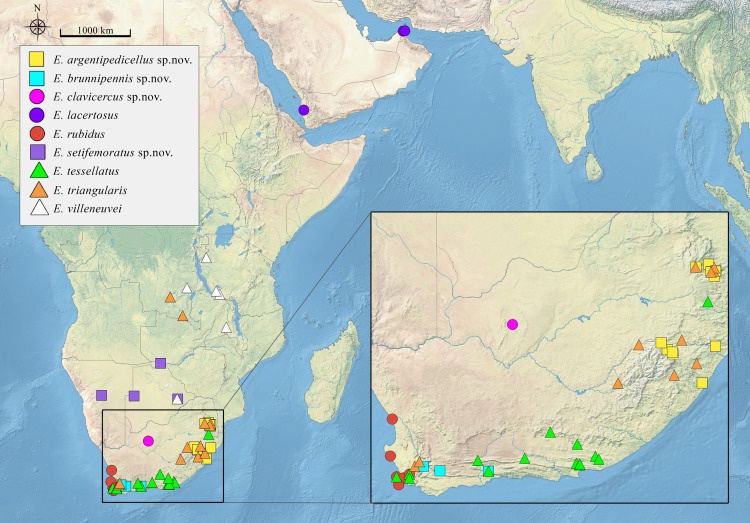
Distribution map of *Eumerus triangularis* species group.

Data on the geographic distribution of analysed species were processed in DivaGis (v7.5) and presented on Fig 1. Phytochoria of Africa considered in this work are after White [42].

Because original descriptions were often very brief and omitted a number of diagnostic characters, and were written in different languages using different terminology, all previously recognised valid species (except recently described *E. lacertosus*) were redescribed according to a standardised format (identical to the descriptive parts of new species) in order to allow comparison for all character states.

### Nomenclatural acts

The electronic edition of this article conforms to the requirements of the amended International Code of Zoological Nomenclature, and hence the new names contained herein are available under that Code from the electronic edition of this article. This published work and the nomenclatural acts it contains have been registered in ZooBank, the online registration system for the ICZN. The ZooBank LSIDs (Life Science Identifiers) can be resolved and the associated information viewed through any standard web browser by appending the LSID to the prefix “http://zoobank.org/”. The LSID for this publication is: urn:lsid:zoobank.org:pub: urn:lsid:zoobank.org:pub:5300D4ED-62B7-465F-9856-8493DF193A74.

The electronic edition of this work was published in a journal with an ISSN, and has been archived and is available from the following digital repositories: PubMed Central, LOCKSS.

### Molecular analysis

Genomic DNA vouchers (S1 Table) are preserved at FSUNS, KMMA and MZH. Genomic DNA was extracted from a single leg using the NucleoSpin Tissue Kit (Macherey-Nagel, Düren), following the manufacturer’s instructions. PCR reactions were undertaken in 25 μl reaction volumes, that contained 1.5 mM MgCl2 in 1 × PCR buffer (Invitrogen), 0.2 mM of each dNTP, 0.2 μM of each primer and 0.5 units of Taq polymerase (Invitrogen). The DNA barcode fragment (or 5’-COI end) of the mitochondrial cytochrome c oxidase subunit I (COI) gene was amplified using primer pair LCO1490 and HCO2198 [43] following Jordaens *et al.* [25]. A fragment of the 3’-end of the COI gene was amplified using primer pair PAT and JERRY [44] and the D2-3 expansion segment of the nuclear ribosomal 28S rRNA gene was amplified using primer pair 28SF2 and 28S3DR [45] following Djan *et al.* [32]. The cytochrome b gene was amplified using primer pair cytb-10933F and cytb-11683R following [44]. The PCR profile was an initial denaturation step of 5 min at 95 °C, followed by 35 cycles of 45 s at 95 °C, 45 s at an annealing temperature of 45 °C (5’-COI end) or 51 °C (3’-COI end, cytb and 28S rDNA) and 1.5 min at 72 °C and ending with a final extension step of 5 min at 72 °C. PCR products were purified using the GFX PCR DNA Purification Kit (GE Healthcare) and diluted in 15 μl of sterile water or using the ExoSap protocol (Invitrogen) following the manufacturer’s instructions. PCR-products were bidirectionally sequenced using the ABI PRISM BigDye Terminator v.3.1 Cycle Sequencing Kit and run on an ABI3130xl Genetic Analyzer. Sequences were assembled in SEQSCAPE v.2.5 (Life Technologies) and inconsistencies were checked by eye on the chromatogram. DNA sequences were submitted to GenBank under the following accession numbers: 5’- end COI (DNA barcodes): PQ570403-PQ570418, 3’- end of COI gen: PQ570423-PQ570437, cytb: PQ570910-PQ570923, and 28rRNA: PQ571004-PQ571015 (S1 Table).

Three datasets were prepared for cluster analysis, one containing DNA barcode sequences (5’end COI) only; one combining all three mtDNA regions, and one combining all three mitochondrial and the nuclear gene fragments. The most appropriate nucleotide substitution model for each dataset was selected using MEGA X [46] using the Akaike Information Criterion [47]. Maximum likelihood (ML) analyses were performed for all three datasets, and partitioned according to gene regions for the latter two datasets. All analyses were rooted on *Eristalis tenax* (Linnaeus, 1758) (DNA voucher 113C03, GenBank Acc. No. S1 Table). Maximum likelihood (ML) trees were constructed in RAxML 8.2.12 [48] through the CIPRES Science Gateway web portal [49] under the general time-reversible (GTR) evolutionary model with gamma distribution (GTRGAMMA). Branch support was estimated with 1000 rapid bootstrap iterations. Uncorrected p-distances were calculated in MEGA X [46].

## Results

### Descriptions and diagnoses

Syrphidae Latreille, 1802
Eristalinae Newman, 1834
Merodontini Edwards, 1915
Genus *Eumerus* Meigen, 1822

Type species: *Syrphus tricolor* Fabricius, 1798, by subsequent designation of Curtis, 1839: 749.

Synonyms:

*Citibaena* Walker, 1856: 124. **Type species**: *Citibaena aurata* Walker, 1856 (monotypy).
*Citibena* Bigot, 1883: 225. Misspelling of *Citibaena* Walker.
*Citabaena* Curran, 1938: 7. Misspelling of *Citibaena* Walker.
*Paragopsis* Matsumura, 1916: 250. **Type species:** *Paragopsis griseofasciatus* Matsumura, 1916 (by original designation).
*Pumilio* Rondani, 1850: 127. **Type species:** *Pumilio delicatae* Rondani, 1850 (monotypy).
*Microxylota* Jones, 1917: 230. **Type species:** *Microxylota robii* Jones, 1917 (by original designation).

#### *Eumerus triangularis* group.

***Diagnosis.*** Medium to large species, with silver pedicel, black or black-reddish elongated abdomen with three pairs of oblique whitish pruinose fasciae on tergites 2–4, incrassate hind femur, and in general a granulated (base of hair with granula not pit) cuticle of head, mesonotum, pleuron, legs and tergites. Pedicel enlarged on the inside, square or elongated, divided in two areas: upper triangular area bare and densely silver-white pruinose (except in *Eumerus brunnipennis* sp. nov. where this area is partly setose), lower are setose and non-pruinose. Eye bare, holoptic in male with an eye contiguity a bit shorter than length of ocellar triangle. Frons densely silver-white pruinose, like film (bare or with sparse hairs along the eye margin in *E*. *argentipedicellus* sp. nov., *E*. *brunnipennis* sp. nov., *E*. *tessellatus*, *E*. *triangularis* and *E*. *villeneuivei*) in male; and completely setose with pruinescence only along the eye margin in female. Face covered with dense, long silver-white microtrichia and hairs. Notopleural suture present. Katepisternum with granulae and hairs additionally present anteriorly and/or ventrally from the setose dorsoposterior corner. Scutum with more or less distinct whitish pruinescence pattern: at least with a trace of three narrow pruinose vittae (lateral vitta becomes a pruinose macula near the end of transverse suture) and tergites 2–4 with a pair of oblique white pruinose fasciae. Hind femur incrassate, with preapical anteroventral flange bearing 7–12 strong spiny setae and posteroventral row of few (at most five) strong spiny setae. Hind tibia with sharp, long anteroventral carina and less sharp, shorter posteroventral carina. Sternite 4 in male posteromedially with incurvation and posterolateral lobes with tuft of hairs (except in *E. clavicercus* sp. nov. where posterolateral lobes are not developed, but with tuft of hairs). Male terminalia: Anterior surstylar lobe very complex, consists of inner setose lobe and apical twisted, microtrichose structure with spiny or finger like protrusion in between; posterior surstylar lobe simple, with strong hairs on inner side; interior accessory lobe of posterior surstylar lobe consists of a larger densely microtrichose part that lies on subepandrial sclerite and a smaller setose lobe; base of epandrium short, consequently cerci retracted up to posterior surstylar lobe. Hypandrium with lateral pruinose area on theca; microtrichia also present near the place where apical end of aedeagus emerges; hamus sickle shaped with microtrichose apex; ejaculatory apodeme large, fan like; aedeagal apodeme with medial keel and lateral wide wings at basis.

#### *Eumerus argentipedicellus* Radenković, Vujić & Grković sp. nov.

urn:lsid:zoobank.org:act:931D890B-9877-45F0-BECB-74FC92F46468

(Figs 1, 2A,B, 3A,B, 4A, 5A, 6A, 7A, 8A, 9A, 10A, 11A, 12A, 13A,B,I)

**Fig 2:**
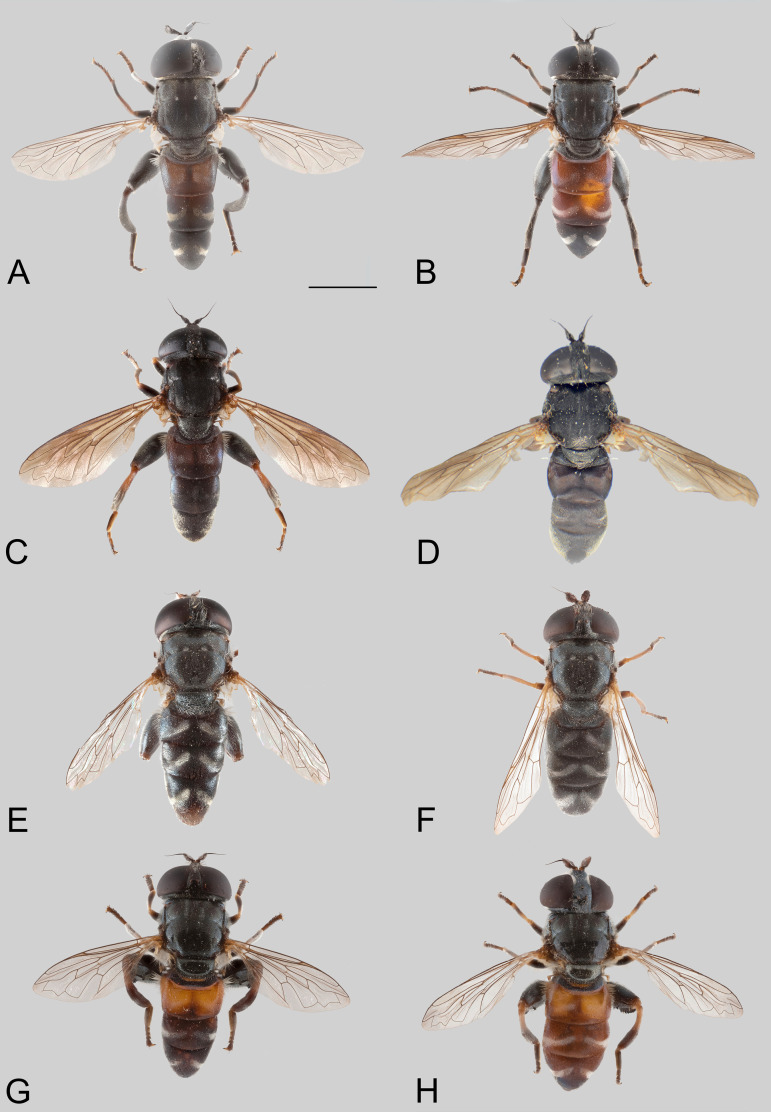
Body, dorsal view. *Eumerus argentipedicellus* Radenković, Vujić & Grković sp. nov.: A, Male. B, Female. *Eumerus brunnipennis* sp. nov. Radenković, Vujić & Grković sp. nov.: C, Male. D, Female. *Eumerus clavicercus* Radenković, Vujić & Grković sp. nov.: E, Male. F, Female. *Eumerus rubidus* Hull, 1964: G, Male. H, Female. Scale: 2 mm.

**Fig 3:**
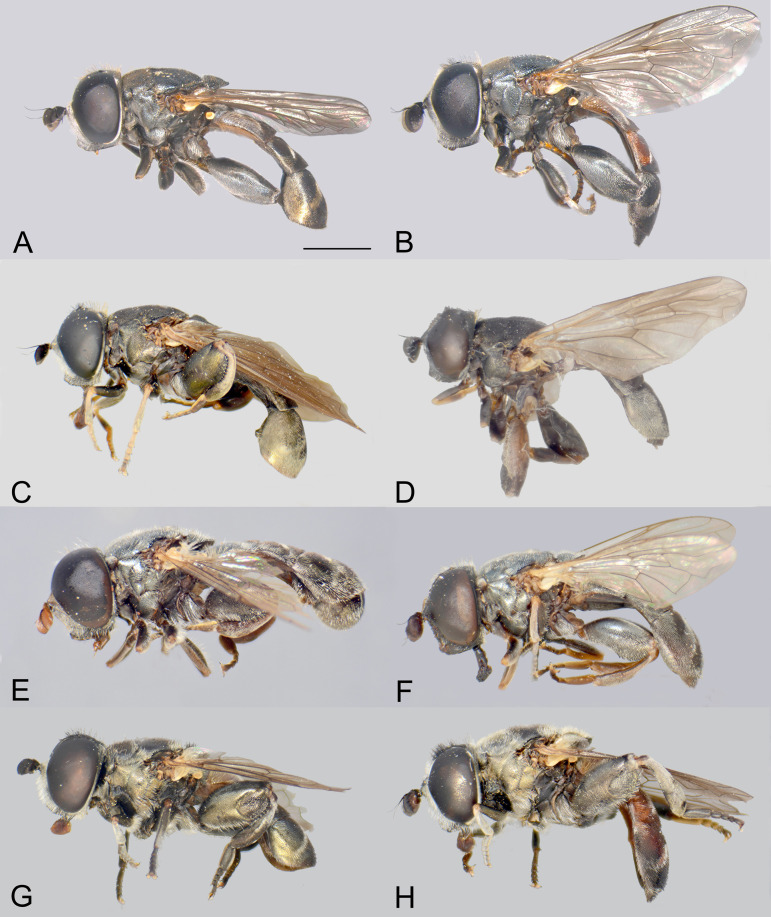
Body, lateral view. *Eumerus argentipedicellus* Radenković, Vujić & Grković sp. nov.: A, Male. B, Female. *Eumerus brunnipennis* sp. nov. Radenković, Vujić & Grković sp. nov.: C, Male. D, Female. *Eumerus clavicercus* Radenković, Vujić & Grković sp. nov.: E, Male. F, Female. *Eumerus rubidus* Hull, 1964: G, Male. H, Female. Scale: 2 mm.

**Fig 4:**
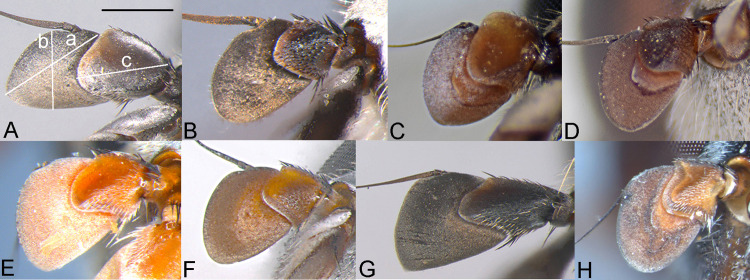
Antenna, male, inner side. A, *Eumerus argentipedicellus* Radenković, Vujić & Grković sp. nov. B, *Eumerus brunnipennis* sp. nov. Radenković, Vujić & Grković sp. nov. C, *Eumerus clavicercus* Radenković, Vujić & Grković sp. nov. D, *Eumerus rubidus* Hull, 1964. E, *Eumerus setifemoratus* sp. nov. Radenković, Vujić & Grković sp. nov. F, *Eumerus tessellatus* Hull, 1964. G, *Eumerus triangularis* Hervé-Bazin, 1913. H, *Eumerus villeneuvei* Hervé-Bazin, 1913. Abbreviations: a – the length of postpedicel; b – the width of postpedicel; c – the length of pedicel. Scale: 0.5 mm.

**Fig 5:**
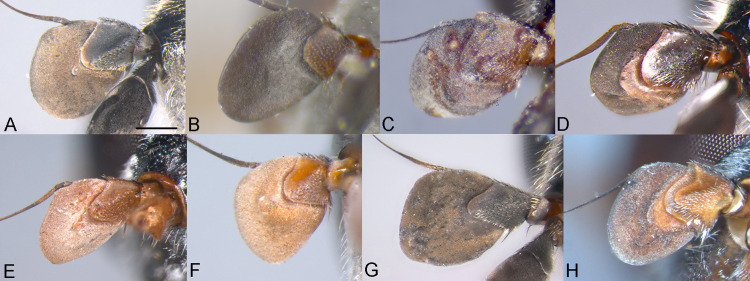
Antenna, female, inner side. A, *Eumerus argentipedicellus* Radenković, Vujić & Grković sp. nov. B, *Eumerus brunnipennis* sp. nov. Radenković, Vujić & Grković sp. nov. C, *Eumerus clavicercus* Radenković, Vujić & Grković sp. nov. D, *Eumerus rubidus* Hull, 1964. E, *Eumerus setifemoratus* sp. nov. Radenković, Vujić & Grković sp. nov. F, *Eumerus tessellatus* Hull, 1964. G, *Eumerus triangularis* Hervé-Bazin, 1913. H, *Eumerus villeneuvei* Hervé-Bazin, 1913. Scale: 0.5 mm.

**Fig 6:**
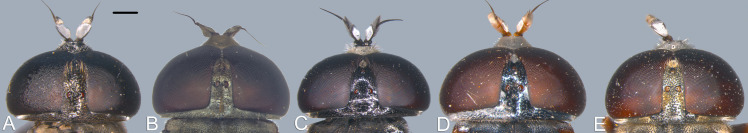
Head, male, dorsal view. A, *Eumerus argentipedicellus* Radenković, Vujić & Grković sp. nov. B, *Eumerus lacertosus* Smit, 2017. C, *Eumerus rubidus* Hull, 1964. D, *Eumerus tessellatus* Hull, 1964. E, *Eumerus villeneuvei* Hervé-Bazin, 1913. Scale: 0.5 mm.

**Fig 7:**
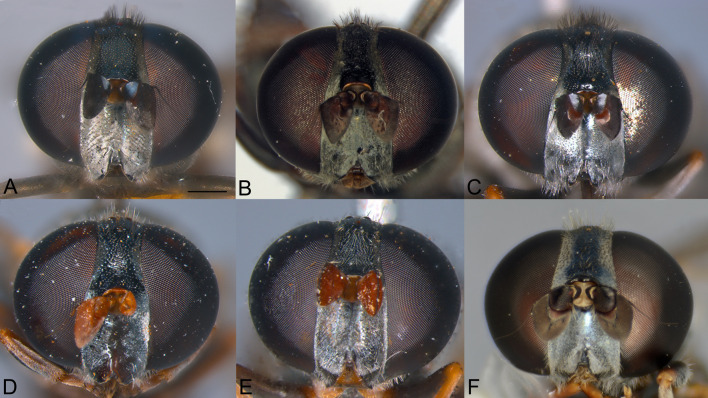
Head, female, anterior view. A, *Eumerus argentipedicellus* Radenković, Vujić & Grković sp. nov. B, *Eumerus lacertosus* Smit, 2017. C, *Eumerus rubidus* Hull, 1964. D, *Eumerus setifemoratus* sp. nov. Radenković, Vujić & Grković sp. nov. E, *Eumerus tessellatus* Hull, 1964. F, *Eumerus villeneuvei* Hervé-Bazin, 1913. Scale: 0.5 mm.

**Fig 8:**
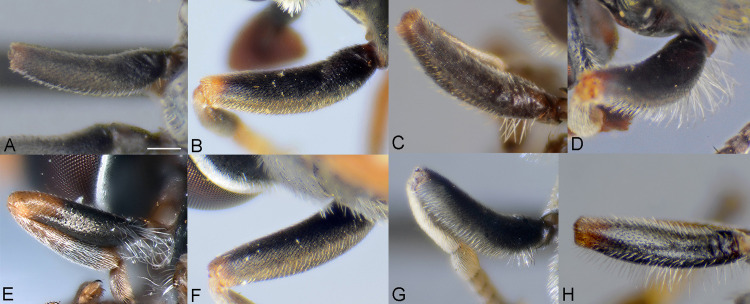
Fore femur, male, dorso-lateral view. A, *Eumerus argentipedicellus* Radenković, Vujić & Grković sp. nov. B, *Eumerus brunnipennis* sp. nov. Radenković, Vujić & Grković sp. nov. C, *Eumerus clavicercus* Radenković, Vujić & Grković sp. nov. D, *Eumerus rubidus* Hull, 1964. E, *Eumerus setifemoratus* sp. nov. Radenković, Vujić & Grković sp. nov. F, *Eumerus tessellatus* Hull, 1964. G, *Eumerus triangularis* Hervé-Bazin, 1913. H, *Eumerus villeneuvei* Hervé-Bazin, 1913. Scale: 0.2 mm.

**Fig 9:**
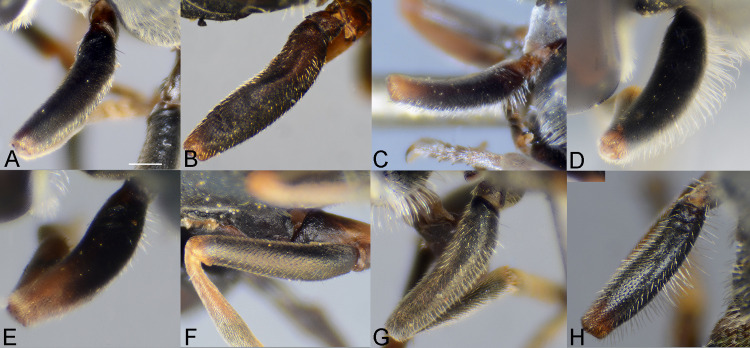
Fore femur, female, dorso-lateral view. A, *Eumerus argentipedicellus* Radenković, Vujić & Grković sp. nov. B, *Eumerus brunnipennis* sp. nov. Radenković, Vujić & Grković sp. nov. C, *Eumerus clavicercus* Radenković, Vujić & Grković sp. nov. D, *Eumerus rubidus* Hull, 1964. E, *Eumerus setifemoratus* sp. nov. Radenković, Vujić & Grković sp. nov. F, *Eumerus tessellatus* Hull, 1964. G, *Eumerus triangularis* Hervé-Bazin, 1913. H, *Eumerus villeneuvei* Hervé-Bazin, 1913. Scale: 0.2 mm.

**Fig 10:**
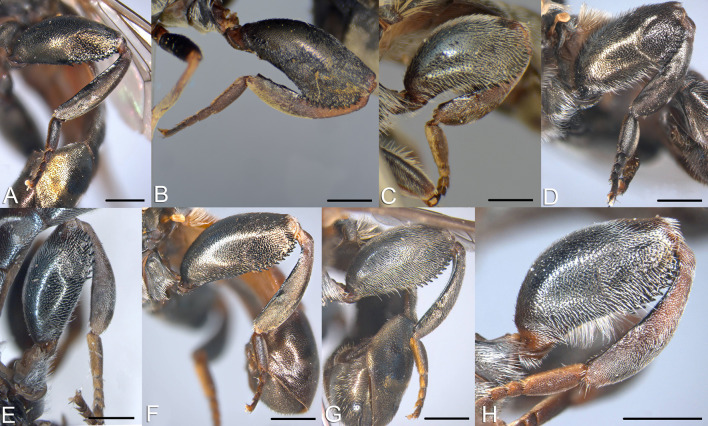
Hind leg, male, lateral view. A, *Eumerus argentipedicellus* Radenković, Vujić & Grković sp. nov. B, *Eumerus brunnipennis* sp. nov. Radenković, Vujić & Grković sp. nov. C, *Eumerus clavicercus* Radenković, Vujić & Grković sp. nov. D, *Eumerus rubidus* Hull, 1964. E, *Eumerus setifemoratus* sp. nov. Radenković, Vujić & Grković sp. nov. F, *Eumerus tessellatus* Hull, 1964. G, *Eumerus triangularis* Hervé-Bazin, 1913. H, *Eumerus villeneuvei* Hervé-Bazin, 1913. Scale: 0.5 mm.

**Fig 11:**
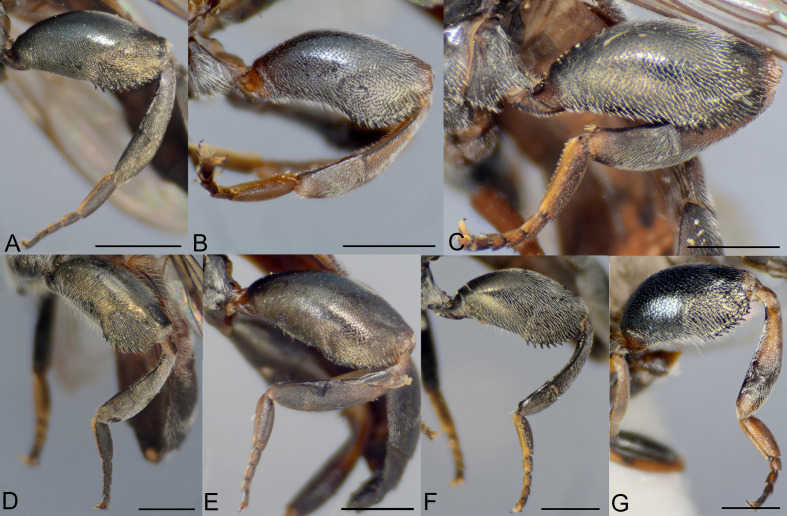
Hind leg, female, lateral view. A, *Eumerus argentipedicellus* Radenković, Vujić & Grković sp. nov. B, *Eumerus clavicercus* Radenković, Vujić & Grković sp. nov. C, *Eumerus setifemoratus* sp. nov. Radenković, Vujić & Grković sp. nov. D, *Eumerus rubidus* Hull, 1964. E, *Eumerus tessellatus* Hull, 1964. F, *Eumerus triangularis* Hervé-Bazin, 1913. G, *Eumerus villeneuvei* Hervé-Bazin, 1913. Scale: 0.5 mm.

**Fig 12:**
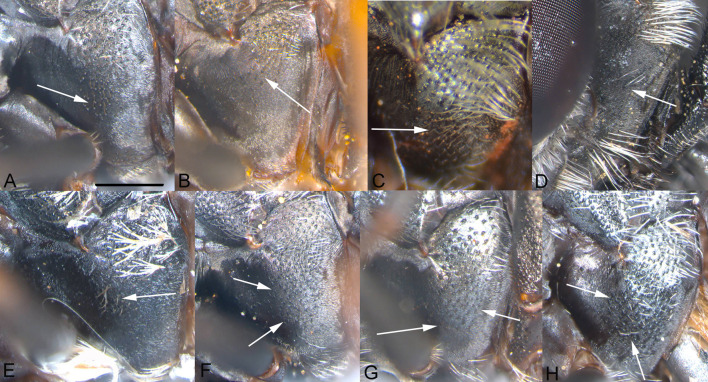
Katepisternum, male. A, *Eumerus argentipedicellus* Radenković, Vujić & Grković sp. nov. B, *Eumerus brunnipennis* sp. nov. Radenković, Vujić & Grković sp. nov. C, *Eumerus clavicercus* Radenković, Vujić & Grković sp. nov. D, *Eumerus rubidus* Hull, 1964. E, *Eumerus setifemoratus* sp. nov. Radenković, Vujić & Grković sp. nov. F, *Eumerus tessellatus* Hull, 1964. G, *Eumerus triangularis* Hervé-Bazin, 1913. H, *Eumerus villeneuvei* Hervé-Bazin, 1913. “Extra” granulation marked with white arrows. Scale: 0.5 mm.

**Fig 13:**
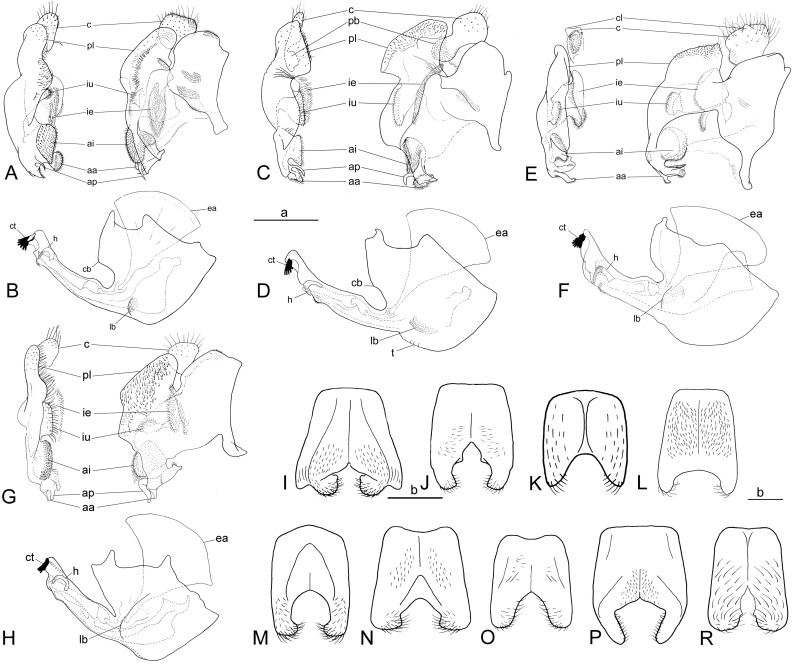
Male terminalia. *Eumerus argentipedicellus* Radenković, Vujić & Grković sp. nov.: A, epandrium, ventral and lateral view. B, hypandrium, lateral view. *Eumerus brunnipennis* sp. nov. Radenković, Vujić & Grković sp. nov.: C, epandrium, ventral and lateral view. D, hypandrium, lateral view. *Eumerus clavicercus* Radenković, Vujić & Grković sp. nov.: E, epandrium, ventral and lateral view. F, hypandrium, lateral view. *Eumerus lacertosus* Smit, 2017: G, epandrium, ventral and lateral view. H, hypandrium, lateral view. Males sternite 4: I, *Eumerus argentipedicellus* Radenković, Vujić & Grković sp. nov. J, *Eumerus brunnipennis* sp. nov. Radenković, Vujić & Grković sp. nov. K, *Eumerus clavicercus* Radenković, Vujić & Grković sp. nov. L, *Eumerus lacertosus* Smit, 2017. M, *Eumerus rubidus* Hull, 1964. N, *Eumerus setifemoratus* sp. nov. Radenković, Vujić & Grković sp. nov. O, *Eumerus tessellatus* Hull, 1964. P, *Eumerus triangularis* Hervé-Bazin, 1913. R, *Eumerus villeneuvei* Hervé-Bazin, 1913. Scale: a (A–H) =  b (I–R) =  500 µm. aa – apical twisted, microtrichose structure of anterior surstyle lobe; ai – inner pilose lobe of anterior surstyle lobe; ap – protrusion between inner lobe and apical structure of anterior surstyle lobe; c – cercus; cb – central buldge of theca; cl – lateral triangular protrusions of cercus; ct – ctenidium; ea – ejaculatory apodeme; h – hamus; ie – smaller pilose lobe of interior accessory lobe; iu – larger densely microtrichose part of interior accessory lobe that lies on subepandrial sclerite; lb – lateral pollinose area of theca; pb – pilose bulge of cercus; pl – posterior surstyle lobe; t –folded theca postero-medially.

***Diagnosis.*** Species with red to brownish-black abdomen (Figs 2A,B, 3A,B) and dark brownish-black antennae. Pedicel innerly enlarged (particularly in male where almost as long as postpedicel), with larger upper silver pruinose area than lower setose area (especially in male) (Figs 4A, 5A, 6A, 7A). Fore (Figs 8A, 8A) and hind legs (Figs 10A, 11A) with short hairs (except on posterior surface of fore femur with one or few moderately long hairs). Wing with cross vein sc-r present. Katepisternum largely covered with setose granulae, not only on dorsoposterior corner, but distributed also deeply towards anterior and ventral margins (Fig 12A). Tergites usually blackish in male (tergite 2 can be paler) (Fig 2A) and paler in female (tergites 2 and 3 reddish) (Fig 2B), with three pairs of oblique white pruinose fasciae on tergites 2–4; hairs black on sternite 4; in male posterolateral lobes of sternite 4 right-angled and provided with a moderately long black hairs of golden reflection. Male terminalia: posterior surstylar lobe in a shape of rounded rectangle covered on inner side with strong hairs situated along diagonal and upper triangle; cercus in a shape quarter of circle (Fig 13A). In female, occiput completely pruinose and vertex posterolaterally as in *Eumerus rubidus* and *E. tessellatus*, from which can be distinguished by completely covered alula with microtrichia, and additionally from *E. rubidus* differs by short setose fore and hind femora. The female can be additionally identified by narrow pruinose vitta along the eye margin (Fig 7A) and the antennal pedicel with upper bare silver microtrichose area slightly larger than lower setose area (Fig 5A). DNA sequences were submitted to GenBank under the following accession numbers: 5’- end COI (DNA barcodes): PQ570403-PQ570418, 3’- end of COI gen: PQ570423-PQ570437, cytb : PQ570910-PQ570923, and 28rRNA: PQ571004-PQ571015 (Supplemental Material: S1 Table).

*Description.* **MALE** *Head*. Eye contiguity ten facets long, without ommatrichia (Fig 6A). Face black with scattered granulae, covered with dense silver microtrichia and whitish hairs (microtrichia scattered only along oral margin); contour slightly concave in lateral view, with oral margin slightly raised. Postpedicel dark brownish-black, of rhomboid shape with the apex protruded ventrally, about 1.5 times longer than wide, ca. 1.2 times longer than pedicel, with outer fossette along anterior margin; pedicel large, with distinctly larger upper silver pruinose area than lower setose area (Figs 4A, 6A). Frons completely covered with dense, silver microtrichia, velvet like film and whitish hairs along eye margin. Vertex black, with scattered strong granulation; covered with white-yellowish hairs except at anterior end which is bare; greyish pruinose on large triangular area in front of anterior ocellus and posterolaterally (a tiny longitudinal medial vitta can be present posteriorly), in addition to lateral whitish pruinose maculae just behind posterior ocelli, along eye margin. Ocellar triangle narrow, isosceles (distance between posterior ocelli 2 times smaller than between anterior and posterior ocelli) (Fig 6A). Occiput narrow, black, completely pruinose, with whitish microtrichia along eye margin (dorsally the most conspicuous close to the shiny dorsal eye corner) and the rest with grey microtrichia; covered with whitish hairs.

*Thorax.* Mesonotum black, densely and distinctly granulate (especially scutellum), covered with short adpressed hairs (Figs 2A, 3A), predominantly yellowish anterior to transverse suture and blackish posterior to suture (including scutellum); strong black setae present at wing basis and on postalar callus; notopleural suture present; whitish pruinescence present on postpronotum, anterior margin of scutum from postpronotum to submedial vitta, laterally in anterior half of scutum and with macula at the end of transverse suture, plus additionally three narrow longitudinal vittae (one medial and two submedial vittae, ending at the posterior 4/5 of scutum), as well as short intraalar vitta after the transverse suture and macula at the posterior end of postalar callus; scutellum with about 20–30 granulae along posterior margin. Pleura dark brownish-black, predominantly covered with white-greyish microtrichia and on the following parts with distinct granulae and whitish hairs: anepisternum, anepimeron, proepimeron, katepisternum and metasternum. Setose granulae on katepisternum are widely distributed, and also present deeply anteriorly and ventrally from hairs on the dorsoposterior corner (Fig 12A). Wing translucent (but with slightly brownish tinge), with yellowish pterostigma, densely microtrichose except the following areas with reduced microtrichia: cell bc, basal end of basal cells r and bm; veins brown except basal parts yellowish; cross-vein sc-r present. Halteres yellowish. Legs predominantly dark brownish-black, except dark orange at fore coxa apically, knees, apex of tibiae, ventral surface of tarsi (in some specimens also base and apex of fore and mid tibiae, and first three tarsomeres dorsally of fore and mid legs paler). Hairs on legs predominantly short and whitish, except black hairs dorsally on fore and mid femora, and apicodorsally on hind femur (Fig 10A), and a few black hairs on tarsomeres apically. Posterior surface of fore femur with few moderately developed long hairs (Fig 8A). Mid femur with long whitish hairs posterodorsally. Hind femur incrassate (2.4 times longer than wide), slightly convex dorsally, covered with short hairs (Fig 10A); its anteroventral flange with ca. 10–11 strong spiny setulae and preapical posteroventral row with ca. 3–4 strong spiny setulae. Hind tibia with sharp anteroventral carina almost reaching the apex (Fig 10A) and less sharp posteroventral carina present in basal third.

*Abdomen.* Abdomen long (1.5 times longer than mesonotum), slender, dark brownish-black (tergite 2 can be paler) (Fig 2A). Tergites finely granulate, except posteriorly on tergite 1 where smooth. Hairs on tergites are short, adpressed except for long silver thick hairs anterolaterally on tergite 2; predominantly black, except on lateral sides where white-yellowish, as well on tergite 1 and on microtrichose bands. Whitish microtrichia present on tergite 1, and on tergites 2–4 as oblique fasciae (almost reaching lateral margins on tergites 2 and 3): V shaped on tergite 2 (with metallic bluish lustre laterally), with concave anterior margin on tergite 3, and the most oblique on tergite 4. Tergite 4 large (almost as long as wide), with lateral sides protruded ventrally and of golden lustre. Sterna 2–3 narrow (about 1/3 width of segment), with greyish microtrichia and scarce, short whitish hairs (and few black ones on sternite 3). Sternite 4 with a black hairs, shallow incurvation posteromedially and posterolateral lobes covered with moderately long black hairs (Fig 13I).

*Male terminalia.* Anterior surstylar lobe very complex, consisting of an inner setose lobus (Fig 13A:ai) with rounded corners and apical twisted microtrichose structure (Fig 13A:aa), plus spiny protrusion in between (Fig 13A:ap); posterior surstylar lobe with the shape of a rounded rectangle on the inner side with strong hairs situated along diagonal and upper triangle (Fig 13A:pl); interior accessory lobe of posterior surstylar lobe consists of microtrichose, elongated part that lies on subepandrial sclerite (Fig 13A:ie) and small rounded strongly setose lobe (Fig 13A:iu); cercus in a shape quarter of a circle. Hypandrium on the wide basal part of theca with lateral microtrichose bulges (Fig 13A:lb) and central bulge anteriorly (Fig 13A:cb); hamus sickle-shaped with microtrichose apex (microtrichia minute) (Fig 13:h); ejaculatory apodeme large, fen-like (Fig 13:ea).

**FEMALE.** Similar to the male except for normal sexual dimorphism and for the following characteristics: frons densely granulate, wide, 0.32 width of head, covered in black hairs above lunulae, the rest with yellowish hairs; pruinescence present only narrowly along eye margin (Fig 7A); vertex predominantly covered in black hairs, especially on ocellar triangle (posteriorly can be with yellowish hairs); postpedicel larger and with more rounded corners, almost as long as wide, and 1.6 times longer than pedicel; border on pedicel that divides upper pruinose area from ventral setose area located slightly below middle (Fig 5A); tergites 2 and 3 reddish, tergite 5 black with black hairs(Fig 2B); sternites 3–5 predominantly covered with black hairs.

Length: body 6–9 mm, wing 5.5–7 mm.

**Material examined.** *Holotype:* **RSA**, Mpumalanga, Barberton, Mountainlands Nature Reserve, -25.791679, 31.143494, 1484 m a.s.l., 1.i.2017, leg. A. Vujić, S. Radenković, N. Veličković & T. Petanidou (1♂: ZA3_027) (FSUNS).

### Paratypes:

**RSA**, Mpumalanga, Gladdespruit river near Asbestos Mine 2530DB, Transvaal 4150 ft, Nelspruit, Kaapsehoop road, 3.xi.1970, leg. Stuckenberg, det. Lyneborg 2006 as unpublished holotype of *Eumerus argenticornis* (1♂: NMSA-DIP 67160) (NMSA); Mpumalanga, Barberton, Heart Beat, -25.821699, 30.952598, 1142m a.s.l., 31.xii.2016, leg. A. Vujić, S. Radenković, N. Veličković & T. Petanidou (4♂♂: ZA3_012, ZA3_014, ZA3_015, ZA3_016; 5♀: ZA3_017, ZA3_018, ZA3_019, ZA3_020; ZA3_021) (FSUNS); 26.xi.2017, leg. A. Vujić, S. Radenković & N. Veličković (5♀: ZA4_095, ZA4_096, ZA4_097, ZA4_098, ZA4_099) (FSUNS); 30.iii.2018, leg. A. Vujić, J. Ačanski, M. Đan & B. Lothrop (2♂♂: ZA5_129, ZA5_134; 1♀: ZA5_135) (FSUNS); Mpumalanga, Barberton, Cynthia Letty Nature Reserve, -25.837735, 30.9483, 1346m a.s.l., 25.ii.2016, leg. A. Vujić, S. Radenković & N. Veličković (1♀: ZA2_172) (FSUNS); Mpumalanga, Barberton, -25.808483, 31.12889, 4.x.2015, leg. A. Vujić et al. (1♂: ZA1_277; 1♀: ZA1_281) (FSUNS), 6.x.2015 (4♂♂: ZA1_282, ZA1_283, ZA1_284, ZA1_288; 1♀: ZA1_290) (FSUNS), 7.x.2015 (3♂♂: ZA1_359, ZA1_360, ZA1_361) (FSUNS); 1.iv.2018, leg. A. Vujić, J. Ačanski, M. Đan & B. Lothrop (4♂♂: ZA5_213, ZA5_214, ZA5_217, ZA5_220; 4♀♀: ZA5_211, ZA5_212, ZA5_216, ZA5_221) (FSUNS); Mpumalanga, Barberton mountain, -26.003219, 31.073652, 2.i.2017, leg. A. Vujić, S. Radenković, N. Veličković & T. Petanidou (1♂: ZA3_030) (FSUNS); Mpumalanga, Barberton mts (geotrail), -28.809046, 31.129585, 1440m, 31.iii.2018, leg. A. Vujić, J. Ačanski, M. Đan & B. Lothrop (4♂♂: ZA5_195, ZA5_196, ZA5_197, ZA5_198) (FSUNS); Mpumalanga, Barberton Nature Reserve, road to Shiyalongubodam, -25.808444, 31.128444, 1420m, 6.x.2015, leg. X. Mengual (1♂: ZFMK DIP 00012226; 7♀♀: ZFMK DIP 00012287, 00012288, 00012289, 00012290, 00012291, 00012293, 00012294) (ZFMK); Mpumalanga, Barberton area, forest road right to Agnes Myn, small valley, -25.837000, 30.947583, 1325 m a.s.l., 7.x.2015, leg. X. Mengual (3♂♂: ZFMK DIP 00012295, 00012296, 00012297) (ZFMK); Mpumalanga, Waterval Boven, -25.649136, 30.342299, 23–24.xi.2017, leg. A. Vujić et al. (1♂: ZA4_081) (FSUNS); KwaZulu-Natal, Drakensberg mountain, Cathedral peak, blue pools, -28.945861, 29.200750, 7.xii.2012, leg. G. Ståhls (1♂: 05772/EU_108, http://id.luomus.fi/GJ.6395) (MZH); KwaZulu-Natal, Drakensberg mountain, Royal Natal, Sunday Falls trail, -28.671186, 28.9506, 1560m, 25.iii.2018, leg. A. Vujić, J. Ačanski, M. Đan & B. Lothrop (3♂♂: ZA5_083, ZA5_084, ZA5_085) (FSUNS), 27.iii.2018 (2♂♂: ZA5_092, ZA5_093; 1♀: ZA5_094) (FSUNS); KwaZulu-Natal, Injisuthi, -29.119451, 29.4364, 10.x.2015, leg. A. Vujić et al. (1♂: ZA1_093) (FSUNS); KwaZulu-Natal, Vryheid Hill Nature Reserve, Overlooking Vryheid town, open savanna/grassland, 29.i–01.ii.2007, leg. G. B. P. Davies (1♂: NMSA-DIP 64968/DNA 1071B01 K. Jordaens RMCA 2016) (NMSA); KwaZulu-Natal, Vernon Crookes Nature Reserve, -30.280833, 30.595556, 450 m a.s.l., mixed grassland, 30.iv.2003, leg. J. G. H. Londt (1♂: NMSA-DIP 64324/DNA 111E07 K. Jordaens RMCA 2016) (NMSA); KwaZulu-Natal, uKhahlamba Drakensberg Park, Monks Cowl N. R., -29.038889, 29.402500, 1358 m a.s.l., montane grassland, 21.i.2006, leg. J. G. H. Londt, (1♂: NMSA-DIP 65155/DNA 114E04 K. Jordaens RMCA 2014) (NMSA).

**Type locality:** South Africa, Mpumalanga.

**Distribution:** Highlands of the eastern parts of South Africa (KwaZulu-Natal and Mpumalanga) (Fig 1).

**Biology:** Flight period: from October to April. Habitat: open savanna and grassland. Phytochoria: Tongaland Pondoland regional mosaic, Afromontane archipelago-like regional centre of endemism.

**Etymology:** The name “*argentipedicellus*”, as an arbitrary combination, is derived from the Latin adjective *argenteus* meaning silver and noun *pedicellus* – second segment of antenna, which refers to the conspicuously silver glistering pedicel of the antenna.

#### *Eumerus brunnipennis* Radenković, Vujić & Grković sp. nov.

urn:lsid:zoobank.org:act:845A51DB-2E94-4EDD-BEF6-9420F6FD6086

(Figs 1, 2C,D, 3C,D, 4B, 5B, 8B, 9B, 10B, 12B, 13C,D,J)

***Diagnosis.*** Black species with black antennae (Figs 2C,D, 3C,D, 4B, 5B). Inner surface of the pedicel almost covered in hairs (contrary to other species of the *triangularis* group), except for an area near the dorsal margin either bare or with scattered hairs and by silver microtrichia. Fore legs with short hairs. Wing with strong brownish coloration (Fig 2C,D); and indication of cross vein sc-r. Setose granulae on the katepisternum are primarily concentrated at the dorsoposterior corner and ventral region, with only a few scattered between these areas and extending toward the anterior end (Fig 12B). Tergites dark brownish-black with three pairs of whitish, oblique microtrichose fasciae on tergites 2–4 (can be less visible). Sternum 4 at the posterior end with a pair of lateral, elongate projections with deep incision between them (Fig 17J). Male terminalia: cercus enlarged, with additional densely short setose bulge (Fig 13E:pb); posterior surstylar lobe stairs like, striated medially, posteroventrally densely setose at inner side (Fig 13E). DNA barcode sequences deposited under accession numbers: XXX-XXX (S1 Table).

**Fig 14:**
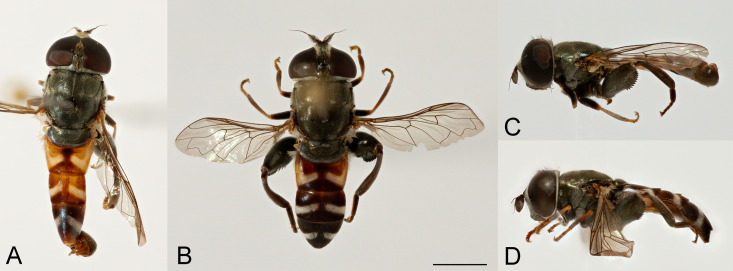
*Eumerus lacertosus* Smit, 2017. Body, dorsal view: A, male. B, female. Body, lateral view: C, male. D, female. Scale: 2 mm.

**Fig 15:**
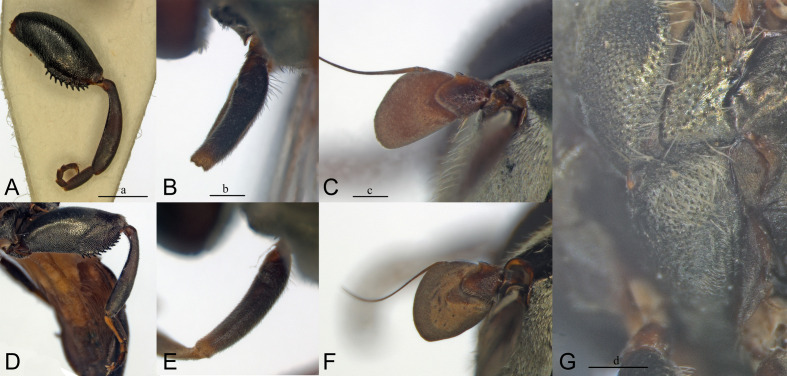
*Eumerus lacertosus* Smit, 2017. A, hind leg, male, lateral view. B, fore femur, male, dorsal view. C, antenna, male, inner side. D, hind leg, female, lateral view. E, fore femur, female, dorsal view. F, antenna, female, inner side. G, katepisternum, male. Scale: a (A, D) =  0.5 mm; b (B, E) =  0.2 mm; c (C, F) =  0.5 mm; d (G) =  0.5 mm.

**Fig 16:**
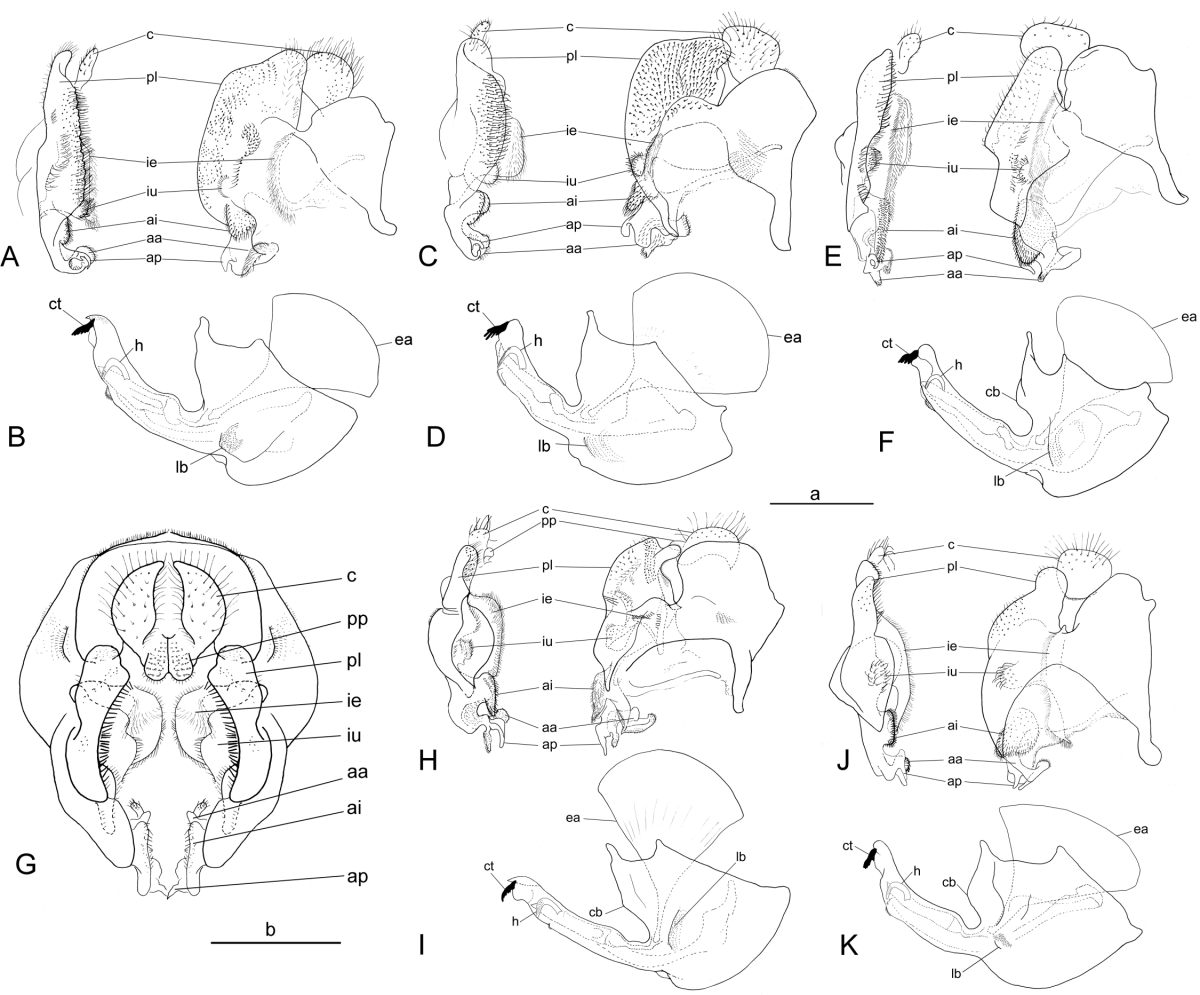
Male terminalia. *Eumerus rubidus* Hull, 1964: A, epandrium, ventral and lateral view. B, hypandrium, lateral view. *Eumerus setifemoratus* sp. nov. Radenković, Vujić & Grković sp. nov.: C, epandrium, ventral and lateral view. D, hypandrium, lateral view. *Eumerus tessellatus* Hull, 1964: E, epandrium, ventral and lateral view. F, hypandrium, lateral view. *Eumerus triangularis* Hervé-Bazin, 1913: G, epandrium, posterior view. H, epandrium, ventral and lateral view. I, hypandrium, lateral view. *Eumerus villeneuvei* Hervé-Bazin, 1913: J, epandrium, ventral and lateral view. K, hypandrium, lateral view. Scale: Scale: a (A–F, H–K) =  b (G) =  500 µm. aa – apical twisted, microtrichose structure of anterior surstyle lobe; ai – inner pilose lobe of anterior surstyle lobe; ap – protrusion between inner lobe and apical structure of anterior surstyle lobe; c – cercus; cb – central buldge of theca; ct – ctenidium; ea – ejaculatory apodeme; h – hamus; ie – smaller pilose lobe of interior accessory lobe; iu – larger densely microtrichose part of interior accessory lobe that lies on subepandrial sclerite; lb – lateral pollinose area of theca; pl – posterior surstyle lobe; pp – pilose protrusion of cercus.

***Description.* MALE.** *Head.* Eyes contiguous for the length of 7–10 facets, without ommatrichia. Face dark brownish-black with scattered granulae, covered with dense silver microtrichia (scattered only medially on raised oral margin and partly laterally within small trapezoid area close to oral margin) and whitish hairs; contour concave in lateral view, with raised oral margin. Antenna dark brownish-black; postpedicel rhombus like with ventrally pointed apex, 1.4 times longer than wide and 1.6 times longer than pedicel, with narrow outer fossette along anterior margin; pedicel on inner side square like, almost entirely covered with hairs except for an area near dorsal margin which is bare or with scattered hairs and silver microtrichia (Fig 4B). Frons completely covered with dense, silver microtrichia like velvet film, and white-yellowish hairs only along eye margin. Vertex black, with dense, distinct granulation, except smooth in front of anterior ocellus and laterally of posterior ocelli; covered with strong black (or mixed black and yellow) hairs anteriorly and predominantly yellow hairs posteriorly; whitish microtrichia present on anterior end in front of anterior ocellus, and posteriorly from the level behind the posterior ocelli till posterior margin. Ocellar triangle narrow, isosceles (distance between anterior and posterior ocelli slightly larger than between posterior ocelli). Occiput black, almost all pruinose, with silver microtrichia along eye margin (dorsally the most conspicuous close to the shiny dorsal eye corner) and the rest with greyish microtrichia; dorsally covered with black, strong, short hairs behind pruinose area, except for a bare patch behind eye corner, and light yellow hairs posteriorly, and whitish hairs laterally.

*Thorax.* Mesonotum blackish (Figs 2C, 3C), except slightly lighter postpronotum posteriorly and postalar callus; densely, distinctly granulate, covered with short adpressed predominantly black hairs (with golden shine), except anteriorly and on postpronotum where white-yellowish; strong setae at wing basis and postalar callus black; notopleural suture present; pruinescence indistinct: on anterior half of postpronotum, anterior margin of scutum from postpronotum to submedial vitta, few microtrichia at the end of transverse suture, trace of three longitudinal vittae, and on anterior margin of scutellum; scutellum with about 30 granulae on posterior margin. Pleura dark brownish-black, predominantly covered with white-greyish microtrichia and on the following parts with distinct granulae and white-yellowish hairs: anepisternum, anepimeron, proepimeron, katepisternum and metasternum. Setose granulae on katepisternum mainly present on dorsoposterior corner and ventrally, and just a few in between and towards anterior end (Fig 12B). Wings with strong brownish coloration (especially anteriorly) (Fig 2C), densely microtrichose, except on the following areas with reduced microtrichia: cell bc, basal end of basal cells R and BM; veins brown; indication of cross-vein sc-r present. Halteres yellowish, slightly darker basally. Legs predominantly dark brownish-black except for orange-yellowish joints and basal half and apex of tibiae, fore and mid tarsi (apical two tarsomeres can be darker), plus ventral surface of hind tarsi. Hairs on legs short (Fig 8B) (except on mid femur posterodorsally long), predominantly white-yellowish, except anteriorly on fore and mid trochanters where black, dorsally and anteriorly on fore and mid femora, basodorsally and apico-posteriorly on hind femur, hind tarsi dorsally, and strong black setae apically on tarsomeres of mid tarsus (and few anteriorly on hind tarsus). Hind femur incrassate, predominantly brownish-black (except for lighter joints) with golden lustrous anteriorly, 2.75 times longer than wide (Fig 10B), preapical anteroventral flange with 10–11 very strong spiny setulae, posteroventral row of 1–3 spiny setulae. Hind tibia with long, sharp anteroventral carina almost reaching the apex (Fig 10B), and less sharp posteroventral carina present only in the basal half, ventro-basal area between two carinae concave.

*Abdomen.* Abdomen long (1.5 times longer than mesonotum), slender, dark brownish-black (except anterior margin of tergite 1 and posterior margin of tergite 4 where are slightly paler) (Fig 2C). Tergites finely granulate, except posteriorly on tergite 1 where are smooth. Hairs on tergites short, adpressed, except for long silver, thick hairs anterolaterally on tergite 2; predominantly black, except for white-yellowish hairs on lateral sides, as well as on tergite 1, parts of the microtrichose bands on tergite 2, and posteriorly on tergite 4. Whitish microtrichia present on tergite 1, and on tergites 2–4 as oblique fasciae, not reaching lateral margins: V shaped and the widest on tergite 2, oblique on tergites 3–4, with concave anterior margin. Tergite 4 large (almost as long as wide), with lateral sides protruded and of golden lustre. Sterna with greyish microtrichia and scarce, short whitish hairs, except on sternite 4 where longer and yellowish. Sternites 2–3 narrow (about 1/3 width of segment); sternite 4 with V shaped incurvation posteriorly, as long as 1/3 of the length of sternite; hairs yellowish, directed from lateral sides to the centre; posterolateral corners covered with tuft of yellow hairs (Fig 13J).

*Male terminalia.* Anterior surstylar lobe complex, consists of two lobes (twisted apical microtrichose part (Fig 13C:aa) and inner setose part (Fig 13C:ai)) with hook like protrusion in between (Fig 13C:ap); posterior surstylar lobe stairs like, striated medially, posteroventrally densely setose at inner side (Fig 13C:pl); interior accessory lobe of posterior surstylar lobe divided in two elongated parts (densely microtrichose that lies on subepandrial sclerite (Fig 13C:ie) and smaller one sparsely setose (Fig 13C:iu)); cercus enlarged, with additional densely short setose bulge (Fig 13C:pb). Hypandrium with two small, lateral bulges (Fig 13D:lb) within the setose area on wide basal part of theca and slightly folded theca posteromedially (Fig 13D:t); hamus sickle shaped (Fig 13D:h), apex with minute microtrichia; ejaculatory apodeme large, fan like (Fig 13D:ea).

**FEMALE.** Similar to the male except for normal sexual dimorphism (Figs 2D, 3D) and for the following characteristics: frons wide (0.3 times width of head), densely granulate, shiny black except for a whitish pruinose vitta along eye margins, covered with mixed black and yellowish hairs; postpedicel larger, oval, ca. 1.4 times longer than wide and ca. 2.5 times longer than pedicel (Fig 5B); sternite 4 predominantly covered with black, short, adpressed hairs.

Length: body 10–11 mm, wing 6–8 mm

### Material examined

*Holotype:* **RSA**, Western Cape, Oudtshoorn District, Moeras Rivier Farm (209), -33.800000, 22.050000, 525 m a.s.l., early September 2007, dry Karoo scrub with flowers, leg. G. Davies, (1♂: NMSA-DIP 75214), (DNA 165F04 K. Jordaens RMCA 2014), (det. J. T. Smit 2016 as *Eumerus* ♂ SA-01) (NMSA).

*Paratypes:* **RSA**, Western Cape, Cape Montagu Pass 5km NE Ashton, 25.ix.1979, 3320CC, Steep rocky hillside, leg. J. G. H. Londt (1♂: NMSA-DIP 43179), (det. Lyneborg 2006 as an unpublished paratype of *Eumerus fuscipennis*) (NMSA); RSA, Western Cape, Cape Karoo Botanic Gardens Worcester, 3319 Cb, 30.xii.1982 – 6.i.1983, Malaise trap, leg. R. M. Miller, (1♀: NMSA-DIP 67097), (det. Lyneborg 2006 as an unpublished paratype of *Eumerus fuscipennis*) (NMSA).

## Type locality

 South Africa, Western Cape.

## Distribution

 South Africa (Western Cape) (Fig 1).

## Biology.

Flight period: September, December, January. Habitat: dry Karoo scrub with flowers. Phytochoria: Cape regional centre of endemism.

## Etymology

 The name “*brunnipennis*”, as arbitrary combination, is derived from the Latin adjective *brunus* meaning brown and the noun *penna –* wing, which refers to the distinctly dark brown wings.

*Eumerus clavicercus* Radenković, Vujić & Grković sp. nov.

urn:lsid:zoobank.org:act:FE08C5EF-0407-4F51-BFAC-851A46A59A81

(Figs 1; 2E,F; 3E,F; 4C; 5C; 8C; 9C; 10C; 11B; 12C; 13E,F, K)

### Diagnosis

Species with black abdomen (Fig 2E,F) except for paler posterior margin of tergite 4 in male and tergite 5 in female, and light-brown/orange antennae. Pedicel large innerly (almost as postpedicel), with upper silver pruinose area much larger than lower setose area (especially in male) (Figs 4C, 5C). Scutum covered with medium long whitish hairs, almost as long as hairs at the beginning of transverse suture. Fore femur with longer but straight hairs posterobasally gradually becoming shorter towards apex (Figs 8C, 9C). Cross vein sc-r present. Katepisternum predominantly covered with setose granulae, not only on dorsoposterior corner, but also distributed deeply towards anterior and ventral margins (Fig 12C). Tergites black, except brownish-orange on tergite 4 posteriorly in male and tergite 5 in female, with three pairs of oblique pruinose fasciae on tergites 2–4. Male terminalia: cercus very specific, with lateral triangular protrusion (Fig 13E:c,cl), laterally giving the appearance of cat ear, almost covered with long thin hairs and ventral side with long, dense, strong microtrichia; posterior surstylar rhomboidal with triangular apex (Fig 13E:pl), covered with strong hairs posteroventrally on inner side; hypandrium stocky (long base, shorter apical part) (Fig 13F). Specific triangular protrusion on cercus distinguishes the male of this species from all other members of the group. Female can be distinguished by moderately long and straight hairs of fore femur posterobasally gradually becoming shorter towards apex (Fig 9C) (in *E. rubidus* basal hairs are curved (Fig 9D) and in *E. setifemoratus* sp. nov. (Fig 9E) and *E*. *triangularis* (Fig 9G) long hairs present only at the base, the rest of hairs very short); and from *E. villeneuvei* can be discerned by the absence of extra long hairs on sternites 2 and 3.

#### Description .

**MALE** *Head*. Eyes contiguous for the length of 7 facets, without ommatrichia. Face dark brownish-black with scattered granulae, covered with dense silver microtrichia (scattered only along oral margin) and whitish hairs; contour slightly concave in lateral view, with oral margin slightly raised. Frons completely silver pruinose, looks like velvet film (without hairs). Vertex black, with scattered granulation and whitish hairs, mostly pruinose, except at the level of, and just behind, the area of ocellar triangle. Ocellar triangle small, equilateral, situated in the middle of the vertex. Antenna light-brown/orange with darker dorsal margin of postpedicel and dark brown arista; postpedicel in a shape of rounded square with slightly protruded apex ventrally, with narrow outer fossette along anterior margin, 1.2 times longer than wide and 1.3 times longer than pedicel; pedicel large, mostly silver pruinose (ridge on median surface of pedicel closer to ventral than to dorsal margin, upper silver pruinose area consequently larger than lower setose area) (Fig 4C). Occiput narrow, black, mostly silver pruinose except shiny at dorsal eye corner; covered with whitish hairs.

*Thorax*. Mesonotum blackish, except lighter on posterior half of postpronotum and postalar callus brown (Fig 2E); densely, distinctly granulate (especially scutellum), covered with moderately long whitish hairs, almost as long as hairs at the beginning of transverse suture; strong black setae present only at wing basis (on postalar callus setae white); notopleural suture present; pruinescence distinct only on anterior half of postpronotum, anterior margin of scutum from postpronotum to submedial vitta, and at the end of transverse suture as a lateral macula, and additionally three indistinct longitudinal vittae (one medial and two submedial vittae, ending at the posterior 4/5 of scutum) present; scutellum with about 30 granulae on posterior margin. Pleura dark brownish-black, predominantly covered with white-greyish microtrichia and on the following parts with distinct granulae and whitish hairs: anepisternum, anepimeron, proepimeron, katepisternum and metasternum. Setose granulae on katepisternum widely distributed, also present anteriorly and ventrally from hairs on the dorsoposterior corner, deeply towards anterior and ventral margin of sclerite (Fig 12C). Wing translucent (but slightly brownish tinge), with yellowish pterostigma, densely microtrichose except on the following areas with reduced microtrichia: cell bc, basal half of basal cells r above and below vena spuria, basal end of cells bm and cup, and anterior third of alula; veins brown, except basal parts where yellowish; cross-vein sc-r present. Halteres yellowish. Legs dark brownish-black, except lighter on following parts: trochanters; base and apex of femora; almost all tibiae (except for a trace of dark subapical ring and on hind tibia with dark anteroventral carina), and tarsi ventrally. Fore trochanter with moderately long whitish hairs; fore femur with longer but straight whitish hairs posterobasally becoming shorter towards apex (Fig 8C); mid femur with long whitish hairs posterodorsally. Hind femur short, incrassate (2 times longer than wide), convex dorsally, with whitish hairs ventrobasally about as long as width of basotarsomere (Fig 10C); its anteroventral flange with 9 strong spiny setae and preapical posteroventral row with 2–3 strong spiny setae. Hind tibia with two long carinae almost reaching the apex (Fig 10C), sharp anteroventral carina with dark brown edge and less sharp posteroventral carina. Hairs on legs predominantly whitish, dorsally on fore and mid femora, and apicodorsally on hind femur. A few black hairs are present at the apical ends of the tarsomeres.

*Abdomen*. Abdomen gradually tapering, 1.5 times longer than mesonotum (Fig 2E). Tergites finely granulate, black except for tergite 4 posteriorly brownish-orange. Whitish pruinescence present on tergite 1 anteriorly, and on tergites 2–4 as wide oblique fasciae, not reaching lateral margins (on tergite 2 is not V shaped, but oblique). Hairs on tergites short, adpressed except for long silver, thick hairs anterolaterally on tergite 2; whitish hairs present on tergite 1, anterolaterally on tergite 2, laterally on tergites 3–4 (and posteriorly on tergite 4), and on pruinose bands on tergites 2–4; the rest are black. Sterna 2–3 narrow (about 1/3 width of segment), with greyish pruinescence and scarce, short whitish hairs, except on sternite 4 where longer. Sternum 4 with an incurvation shaped as a very wide U (as long as 1/3 of the length of sternite), without distinct posterolateral lobes but with tuft of yellowish hairs posterolaterally (Fig 13K).

*Male terminalia*: Anterior surstylar lobe complex, consists of inner trapezoidal strongly setose lobus (Fig 13E:ai) and apical twisted, microtrichose structure (Fig 13E:aa) (without spiny protrusion in between); posterior surstylar rhomboidal with triangular apex (Fig 13E:pl), covered in strong hairs on the posteroventral area of the inner side; interior accessory lobe of posterior surstylar lobe consists of elongated part that lies on minis (with very long microtrichia along margin and apically) (Fig 13E:ie), and upper small rounded strongly setose part (Fig 13E:iu); cercus very specific, with lateral triangular protrusions (Fig 13E:c,cl), all covered in long thin hairs and additionally on ventral side by long, dense, strong microtrichia. Hypandrium stocky (long base, shorter apical part), with rounded apex and lateral folds behind ctenidium (Fig 13F); microtrichose near the place where apical end of aedeagus emerge; basal part has a shorter anterior end, featuring three distinct bulges (centrally the biggest); aedeagal apodeme widen basally in a shape of semicircular fan; hamus sickle-shaped with microtrichose apex (microtrichia minute and somewhat longer at the corner); ejaculatory apodeme large, fen like (Fig 13F:ea).

**FEMALE**. Similar to the male except for normal sexual dimorphism (Figs 2F, 3F) and for the following characteristics: frons wide (0.3 width of head), densely granulose, shiny black except for whitish pruinose vitta along eye margins, covered in thin whitish hairs; vertex without microtrichia in front of anterior ocellus; postpedicel slightly larger and darker, ca. 1.1 times longer than wide and 1.2 times longer than pedicel (Fig 5C); tergite 5 posteriorly lighter.

Length: body 7.5–8 mm, wing 5.5–6 mm

### Material examined

*Holotype:* **RSA**, Northern Cape, 26 mls North of Postmasburg, x.1939, Museum staff (1♂: SAM-DIP A014339), (IMAGED LAS 49 SAMC 2019) (det. Lyneborg 2006 as an unpublished holotype of *Eumerus funebris*) (SAMC).

*Paratype:* 1♀, same data as holotype (det. Lyneborg 2006 as an unpublished paratype of *E. funebris*).

**Type locality:** South Africa, Northern Cape.

**Distribution:** South Africa (Northern Cape) (Fig 1).

**Biology:** Flight period: October. Habitat: savanna. Phytochoria: Kalahari-Highveld regional transition zone.

**Etymology:** The name “***clavicercus***”, as arbitrary combination, is derived from Latin noun *clavus* meaning excrescence, and *cercus* – sensory appendage at the end of the body, which refers to the specific triangular protrusion on cercus.

#### *Eumerus lacertosus* Smit, 2017.

(Figs 14; 6B; 7B; 15; 13G,H,L)

***Diagnosis.*** Species with reddish-brown abdomen (Fig 14) and brown antenna. Ridge on the median surface of pedicel at the same distance from dorsal and ventral margin; consequently upper bare silver microtrichose area and lower setose area that is divided by the ridge are almost the same size (Fig 15C,F). Fore legs with short hairs, except on the basal part of fore femur posteriorly where longer (Fig 15B,E). Cross vein sc-r present. Setose granule on katepisternum extended deeply towards the anterior and ventral margin (Fig 15G). Tergites redish-brown to various extent, with three pairs of oblique pruinose fasciae on tergites 2–4. Male terminalia: posterior surstylar lobe in a form of short stairs (Fig 13G:pl), innerly densely setose including the anterior angle; cercus semicircular (Fig 13G:c). Very similar species to *E. tessellatus* from which can be distinguished by the tuft of longer hairs on fore leg basally (Fig 15B,E), which are absent in *E*. *tessellatus* (Figs 8F, 9F); small ocellar triangle with posterior ocelli very close to each other, located in the anterior half of vertex (Fig 6B) (in *E*. *tessellatus* ocellar triangle larger with posterior ocelli located in the posterior half of vertex) and male terminalia features (in *E. tessellatus* posterior surstylar lobe in a form of longer stairs, innerly densely setose, except on anterior angle where bare; cercus in a shape of upright rectangle).

Length: body 10–11 mm, wing 6–7 mm (n =  10).

***Material examined.*** Holotype, ♂. Label 1: “YEMEN, 12 km NW of/ Manakhah MT/ 15°05’N 43°12’E/ 24.vi–4.viii.2003/Leg. A. Van Harten”. Label 2: “HOLOTYPE/ Eumerus ♂/ lacertosus sp.n./ Det J.T. Smit 2014”. The holotype is in good condition and is deposited in RMNH.

Paratypes: 2♂♂, 4♀♀, same locality as holotype, vii.2001–x.2003.

Additional material: UAE: Wadi Maidaq, 1♂, 14–25.i.2006. Wadi Wurayah, 2♀♀, iv.2005–iii.2007 (RMNH).

**Type locality:** Yemen.

**Distribution:** Yemen and UAE (Fig 1).

**Biology:** Flight period: January, from June to August. Habitat: possibly xeromorphic shrublands.

#### *Eumerus rubidus* Hull, 1964.

(Figs 1; 2G,H; 3G,H; 4D; 5D; 6C; 7C; 8D; 9D; 10D; 11D; 12D; 13M; 16A,B)

***Diagnosis.*** Species with reddish-brown tergites (Fig 2G,H) and three pairs of oblique microtrichose fasciae, generally with longer hairs (on face, scutum, legs and abdomen). Face with a straight contour and long whitish hairs extending mouth edge. Antenna brownish; pedicel with a a smaller, setose lower area and a slightly larger, silvery-white pruinose upper area (Figs 4D, 5D). Fore legs with long, curved hairs on the ventral side of the fore trochanter and on the basal third of the fore femur, both ventrally and posteriorly (gradually becoming shorter towards apex of fore femur) (Figs 8D, 9D); mid femur besides long hairs posterodorsally present in all members of the group, also with medium long hairs ventrally; hind femur with moderately long hairs ventrally and posteroventrally (Figs 10D, 11D). Scutum covered with medium long hairs almost as long as those at the beginning of transverse suture; notopleural suture present. Cross vein sc-r absent (or with indication). Katepisternum besides setose granulae on dorsoposterior corner and ventral end also with an isolated group of 6–8 setae near the centre of sclerite (Fig 12D), which are not easily observable. Male terminalia: posterior surstylar lobe large, of rhomboid shape with triangular apex (Fig 16A:pl), striated on outer side medially, almost covered with strong hairs on inner side; cercus rounded. Besides male terminalia features, *Eumerus rubidus* can be distinguished from other species of the group with redish-brown tergites, (*E. tessellatus* and female of *E. argentipedicellus* sp. nov.) by long and curved hairs on fore leg (fore trochanter ventrally and basal third of fore femur ventrally and posteriorly) and by katepisternum with a group of hairs near its centre (Fig 12D). It can be distinguished from the related species *E. setifemoratus* sp. nov. by length of hairs in the apical half of fore femur (Figs 8D, 9D) (longer in *E*. *rubidus* and short in *E. setifemoratus* sp. nov.) and by the colour and shape of antenna (Figs 4D, 5D) (brownish-black in *E*. *rubidus* and orange-yellow in *E. setifemoratus*, and in male with upper bare area of pedicel slightly larger than the lower setose area in *E*. *rubidus* contrary to *E. setifemoratus* sp. nov. with almost equally sized areas). DNA barcode sequences deposited under accession numbers: XXX-XXX (S1 Table).

***Redescription.* MALE** *Head*. Eye without ommatrichia (Fig 6C), in male contiguous for the length of 8–9 facets. Face with a straight contour and long whitish hairs extending mouth edge. Frons silver pruinose like velvet film, without hairs. Vertex black, mostly shiny, except shortly pruinose at anterior end and indistinct trace of pruinose lateral maculae near the eye margin behind posterior ocelli, finely granulate, covered in predominantly black hairs, except on the posterior end where whitish. Ocellar triangle isosceles, situated in anterior half. Antenna brownish (can be more paler near the border between pedicel and postpedicel, especially on inner side; postpedicel can be light brown/orange in female); pedicel with a longitudinal ridge that separates a smaller, setose lower area from a slightly larger, bare, silvery-white pruinose upper area (Fig 4D). Occiput moderately wide medio-dorsally and narrow laterally, black shiny dorsally, and with silver pruinescence along eye margin (distinct pruinose fascia along posterior eye margin near the dorsal eye corner can be present), except at dorsal eye corner where shiny. *Thorax*. Mesonotum black (except at posterior end of postpronotum and on the postalar callus where paler) (Fig 2G); finely granulate, covered with medium long hairs (almost long as those at the beginning of transverse suture); hairs predominantly black, except on anterior end, laterally until wing basis, at and near the postalar callus and in front of scutellum where whitish, and on scutellum with mixed black and whitish hairs (hairs in female generally longer and on scutellum with predominantly whitish hairs); strong setae at wing basis and postalar callus black; notopleural suture present; pruinescence present on anterior margin of scutum and scutellum, and as three longitudinal vittae (one medial and two submedial vittae, ending at the posterior 4/5 of scutum) plus macula at the end of transverse suture; scutellum with about 30–35 granulae on posterior margin. Katepisternum besides setose granulae on dorsoposterior corner and ventral end also with an isolated group of 6–8 setae near the centre of sclerite, which are not easily observable (Fig 12D). Wing brownish with yellowish pterostigma, densely microtrichose except for an almost bare cell bc and large bare anterior area of alula; veins brown except basal parts where yellowish; cross-vein sc-r absent (or with indication). Halteres yellowish. Legs dark brownish-black except joints and ventral surface of tarsi where orange-yellowish. Fore legs with long and curved whitish hairs on the following parts: fore trochanter ventrally and fore femur in basal third ventrally and posteriorly (gradually becoming shortly towards apex of fore femur) (Fig 8D); mid femur besides long hairs posteriorly present in all members of the group, also with medium long hairs ventrally; hairs in the leg whitish, except for short black hairs anterodorsally on fore and mid trochanters, dorsally and anteriorly on fore and mid femora, basodorsally and apically on hind femur, apical third of anteroventral carina on hind tibia, as well with a few black hairs apically on tarsomeres. Fore tarsi widen, anteriorly tarsomeres with slightly protruded apex; basotarsomere of hind tarsi slightly thickened. Hind femur incrassate, 2 times longer than wide (Fig 10D), preapical anteroventral flange with 8–9 strong spiny setae, and posteroventrally surface with 2–3 strong spiny setae, covered with moderately long hairs ventrally and posteroventrally, whitish, except in male behind the anteroventral flange where black; hind tibia with sharp anteroventral carina almost reaching the apex (Fig 10D), and less sharp posteroventral carina ending at basal half. *Abdomen*. Tergites orange-brown, gradually tapering (Fig 2G). Tergites 1 and 4 predominantly dark brown (except posterior margin of tergite 1, and in some specimens on tergite 4 anteriorly and/or posterior margin where paler), tergites 2 and 3 reddish-brown to a various extent. Whitish pruinescence present on tergite 1 anteriorly, and as oblique pruinose fasciae on tergites 2–4: fasciae on tergite 2 short (not V shaped), by far not reaching lateral margin; those on tergites 3 reaching lateral margin (anterior margin of fasciae almost straight, not concave), and on tergite 4, the most oblique and almost reaching the lateral margin. Hairs on tergites short, adpressed except for long silver, thick hairs anterolaterally on tergite 2; predominantly black, except for whitish hairs on tergite 1, on microtrichose bands, laterally on tergite 2 and posterolaterally on tergites 3–4. Sterna narrow, with greyish microtrichia and scarce, whitish hairs. Sternum 4 in male with semicircular incurvation posteriorly (0.4 of the length of sternite) and posterolateral corners covered with tuft of yellow hairs directed from lateral sides to the centre (Fig 13M). *Male terminalia*: Anterior surstylar lobe complex, consists of inner rounded setose lobus (Fig 16A:ai) and apical twisted, microtrichose structure (Fig 16A:aa) with curved bifurcated protrusion in between (Fig 16A:ap); posterior surstylar lobe large, of rhomboid shape with triangular apex (Fig 16A:pl), striated on outer side medially, almost covered with strong hairs on inner side; interior accessory lobe of posterior surstylar lobe consists of microtrichose, elongated part that lies on minis (Fig 16A:ie) and upper small rounded strongly setose part (Fig 16A:iu); cercus in a shape quarter of a circle (Fig 16A:c), apically not pigmented, the rest dark brown. Hypandrium with apex curved upwards (Fig 16B), microtrichose near the place where apical end of aedeagus emerge; on the wide basal part with small lateral setose bulges (Fig 16B:lb); hamus sickle-shaped with microtrichose apex (microtrichia minute) (Fig 16B:h); ejaculatory apodeme large, fen like (Fig 16B:ea).

**FEMALE.** Similar to the male except for normal sexual dimorphism (Figs 2H, 3H) and for the following characteristics: frons wide (0.3 width of the head), shiny, except along eye margin where whitish pruinose, pruinescence being the widest at about half of the length; covered with black hairs above lunulae, the rest predominantly whitish; vertex non-pruinose, shiny; antenna brownish; postpedicel can be light brown/orange; setose lower area and bare, silvery-white pruinose upper area of pedicel almost of the same size; postpedicel ca. 1.2 times longer than wide, and 1.4 longer than pedicel (Fig 5D). Abdomen wider than in male; predominantly reddish coloured; tergite 3 ca. two times wider than long (Fig 2H).

Length: body 8–10 mm, wing 6–7 mm.

**Material examined.** *Holotype:* **RSA**, Western Cape, Cape Province, Cape Peninsula, Hout Bay, Skoorsteenkop, 2.ii.1951, No. 166 (Insect trap), (Swedish South Africa Expedition, 1950–1951, leg. Brinck & Rudebeck) (det. F. M. Hull *Eumerus rubidus* Hull) (Zool. Mus. Lund Sweden, Syrphidae, type no. 3019:1), (ZML. 396 2004) (1♀: MZLU 098 2019), (Photo 2019 by MZLU) (MZLU).

*Additional material*: **RSA.** Western Cape, West Coast N. P., Duinepos, 33°11’39.6“S, 18°08’18.3”E, 22–23.x.2012, 0 m a.s.l., Malaise traps coastal Fynbos, leg. A. H. Kirk-Spriggs (1♂: BMSA(D) 40649), (Entomology Dept. National Museum P. O. Box 266, Bloemfontein 9300), (DNA 110A08 K. Jordaens RMCA 2014), (det. K. Jordaens & M. de Meyer as *Eumerus* sp.) (BMSA); RSA, Western Cape, Wolfgat Nature Reserve, S Coast Strandveld, 34°04’S, 18°39’E, 5.ix.1995, leg. S van Noort (1♂: NMSA-DIP 67051), (Lyneborg det. 2006 as *Eumerus rubidus* Hull) (NMSA); RSA, Western Cape, Table Mountain Nat. Park, Cape of Good Hope 20m, Cape of Good Hope area, coastal fynbos, 10.x.2006, 34°20’42.0”S, 18°27’45.3”E, leg. J. G. H. Londt, (1♂: NMSA-DIP 65331), (DNA 1071B08 K. Jordaens RMCA 2016) (NMSA); RSA, Western cape, Cape P., Brandfontein Reserve, 34°46’S, 19°52’E, 16–18.x.1992, Strandveld Malaise trap, leg. H. G. Robertson (1♂: NMSA-DIP 67052), (Lyneborg det. 2006 as *Eumerus rubidus* Hull); RSA, Western Cape, Papendorf Olifants Mouth, 31°43’01.2”S, 18°12’22.2”E, Vegetated sand dunes near ocean 10 m a.s.l., 29.ix.2009, leg. J. G. H. Londt & T. Dikow (1♀: NMSA-DIP 65110), (DNA 111F02 K. Jordaens RMCA 2014) (NMSA); RSA, Western Cape, Stellenbosch, 25.ix.1920 (1♂: NMSA-DIP 48032), leg. Brauns (NMSA).

**Type locality:** South Africa, Western Cape.

**Distribution:** South Africa (Western Cape) (Fig 1).

**Biology:** Flight period: September–October, January. Habitat: coastal fynbos. Phytochoria: Cape regional centre of endemism, Karoo Namib regional centre of endemism.

**Note:** Species was described based on a single female.

#### *Eumerus setifemoratus* Radenković, Vujić & Grković sp. nov.

urn:lsid:zoobank.org:act:1E638DA4-9C08-4B7D-B1C6-5A4209E14F39

(Figs 1, 4E, 5E, 7D, 8E, 9E, 10E, 11C, 12E, 13N, 16C,D, 17A,B, 18A,B)

**Fig 17:**
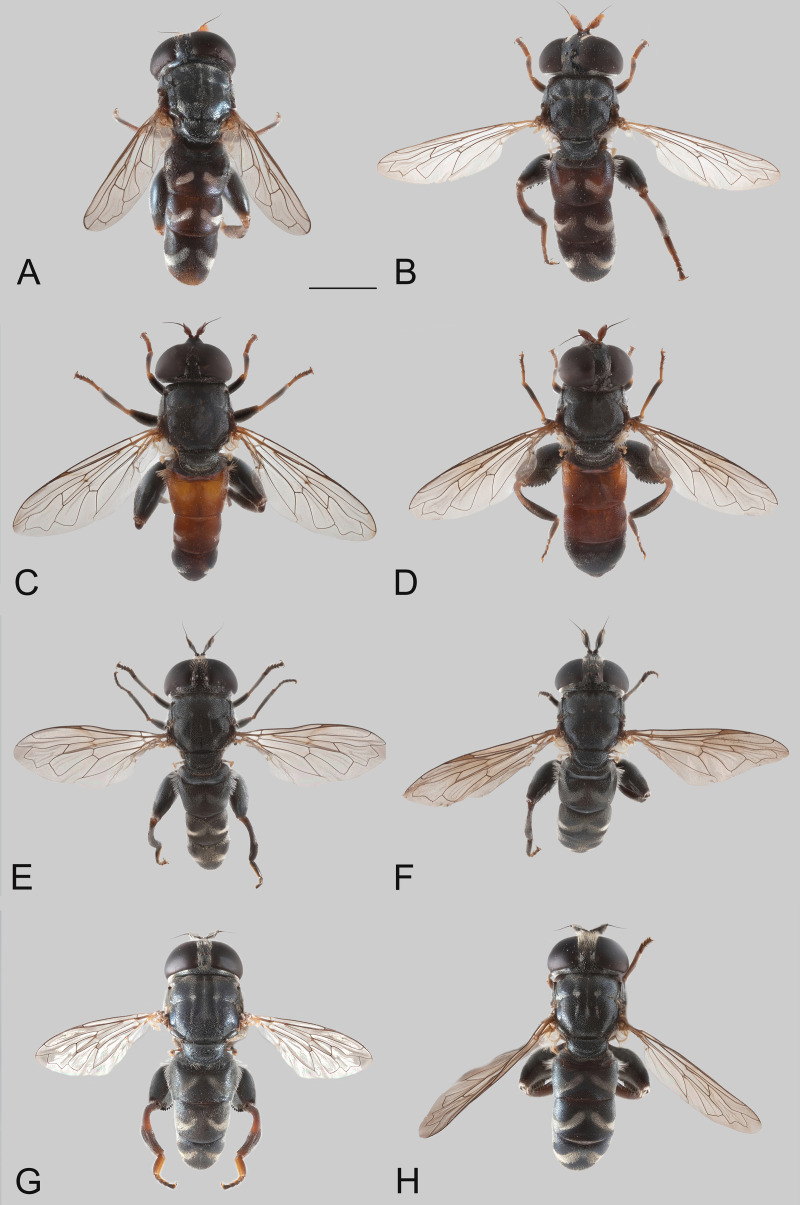
Body, dorsal view. *Eumerus setifemoratus* sp. nov. Radenković, Vujić & Grković sp. nov.: A, Male. B, Female. *Eumerus tessellatus* Hull, 1964: C, Male. D, Female. *Eumerus triangularis* Hervé-Bazin, 1913: E, Male. F, Female. *Eumerus villeneuvei* Hervé-Bazin, 1913: G, Male. H, Female. Scale: 2 mm.

**Fig 18:**
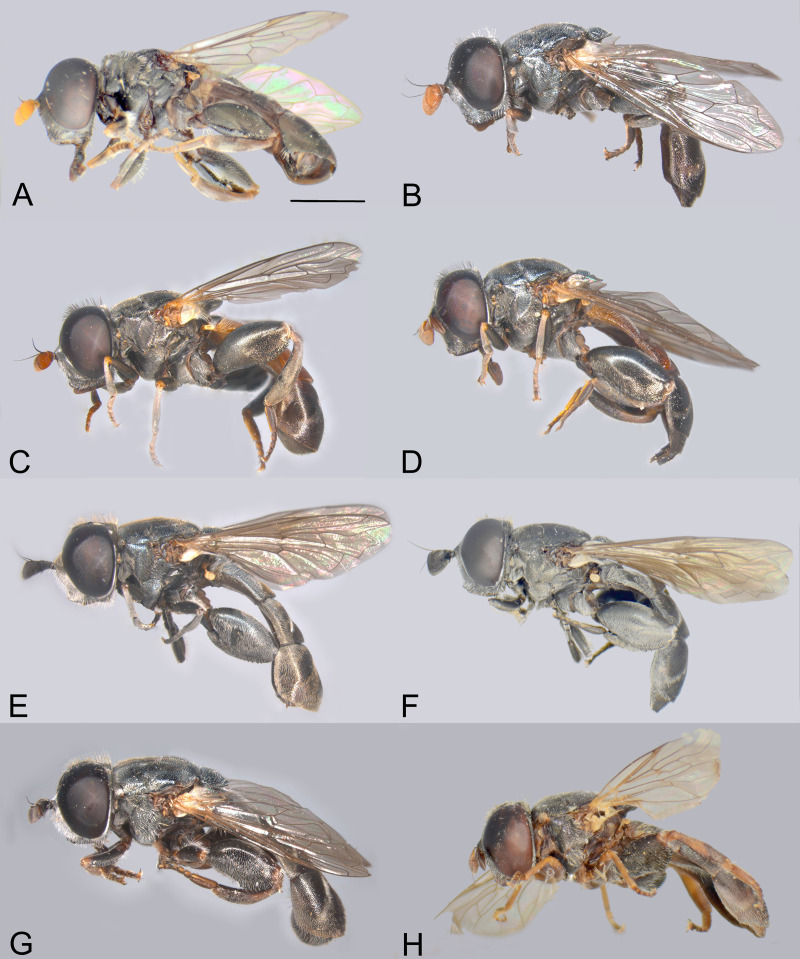
Body, lateral view. *Eumerus setifemoratus* sp. nov. Radenković, Vujić & Grković sp. nov.: A, Male. B, Female. *Eumerus tessellatus* Hull, 1964: C, Male. D, Female. *Eumerus triangularis* Hervé-Bazin, 1913: E, Male. F, Female. *Eumerus villeneuvei* Hervé-Bazin, 1913: G, Male. H, Female. Scale: 2 mm.

***Diagnosis.*** Species with brownish-black or reddish-brown tergites and yellow-orange antenna (Figs 17A,B, 18A,B). Pedicel with bare silver microtrichose area and ventral setose area of almost the same size (Figs 4E, 5E). In males, the fore trochanter and the fore femur, particularly in the basal third, are covered with long, whitish hairs that curve towards the tip (female with straight hairs, shorter on fore trochanter than in basal part of fore femur), the rest of hairs on fore legs short (Fig 8E, 9E). Wing translucent, except for yellowish pterostigma; cross vein sc-r absent. Hairs on scutum short. Katepisternum, except on dorsoposterior corner and ventrally, also with a few setose granulae towards the anterior end (Fig 12E). Tergites 2–4 with three pairs of distinct, oblique, wide, silver pruinose fasciae. Male terminalia: posterior surstylar lobe almost all covered in strong hairs on inner side, in a shape of wide rectangle with rounded protruded setose end towards cercus (Fig 16C:pl); cercus in a shape of quarter circle (Fig 16C:c). From the most related species of the group, *Eumerus rubidus*, except for the male terminalia features, can also be distinguished by length of hairs in the apical half of fore femur (Fig 8E) (short in *E. setifemoratus* and longer in *E*. *rubidus*) and by the color and shape of antenna (Fig 4E) (orange-yellowish in *E. setifemoratus* and brownish-black in *E*. *rubidus*; and in male with upper bare area of pedicel slightly larger than the lower setose area in *E*. *rubidus* contrary to *E. setifemoratus* with almost equally sized both areas). DNA barcode sequences deposited under accession numbers: XXX-XXX (S1 Table).

***Description.* MALE.** *Head.* Eyes contiguous for the length of 8–10 facets, bare. Face dark brownish-black with scattered granulae, covered with dense silver microtrichia (scattered only medially on slightly raised oral margin and partly laterally within a small trapezoid area close to oral margin) and whitish hairs; contour almost straight. Antenna predominantly yellowish-orange; arista orange basally, the rest brown; postpedicel yellow-orange (can be with dark dorsal margin), rhombus-like with apex ventrally pointed, 1.4 times longer than wide and 1.3–1.5 times longer than pedicel, outer fossette narrow, along anterior margin; pedicel on inner side square like, ridge on the median inner surface of pedicel at the same distance from dorsal and ventral margins, consequently upper bare silver microtrichose area and lower setose area are almost the same size (Fig 4E). Frons completely covered with dense, silver microtrichia like velvet film, without hairs. Vertex black, with almost parallel lateral sides and scattered granulation; covered with scarce and thin predominantly black hairs medially and whitish hairs anteriorly and posteriorly; whitish microtrichia present anteriorly, and a large macula posterolaterally behind posterior ocellus and tiny longitudinal posteromedial vitta. Ocellar triangle narrow, isosceles, situated in the anterior half of the vertex. Occiput narrow, black-bluish, shiny, except laterally where with silver microtrichia and dorsally along eye margin till conspicuous semi-circle macula located near the shiny dorsal eye corner; covered with whitish hairs.

### Thorax.

Mesonotum blackish (postalar callus can be brownish) (Fig 17A); densely, distinctly granulate, covered with short adpressed predominantly whitish hairs, except for transversal fascia of mostly black hairs at the wing level on scutum and a few black hairs medially on scutellum; hairs on scutum mostly directed posteriorly, except on posterior fourth where directed anteriorly; strong setae at wing basis black and on postalar callus brown (can be scarce); notopleural suture present; whitish pruinescence present on: postpronotum, five longitudinal vittae (one medial reaching posterior 3/4; two submedial vittae starting anteriorly as triangles, then continuing as weak vitta until transverse suture where it turns in the macula, again continuing as a vitta and ending at about posterior 3/4; and two intraalar vittae starting at transverse suture as a macula, and ending above postalar callus, also as a macula), and additionally on posterior margin of scutum and anterior margin of scutellum; scutellum with about 25 granulae on posterior margin. Pleura dark brownish-black, predominantly covered with white-greyish microtrichia and on the following parts with distinct granulae and whitish hairs: anepisternum, anepimeron, proepimeron, katepisternum and metasternum. Setose granulae on katepisternum, except on dorsoposterior corner and ventrally, also present towards anterior end. Wing translucent, with yellowish pterostigma, densely microtrichose, except on the following areas with reduced microtrichia: cell bc, basal half of basal cells r above and below vena spuria, basal end of cells bm and cup, and small bare area near to anterobasal corner of alula; veins brown, except basal parts where yellowish; cross-vein sc-r absent. Halteres yellowish, slightly darker basally. Legs predominantly dark brownish–black, except orange-yellowish on following parts: apex of fore and mid coxae, fore and mid trochanters, basal end and apex of femora, basal half and apex of tibiae, first three tarsomeres (dorsal surface of hind tarsus can be darker). Hairs on legs long and curved on fore trochanter ventrally and posteriorly, as well as on basal third of fore femur (Fig 8E), plus long and straight on mid femur posterodorsally; the rest hairs short; predominantly whitish, except for a few black hairs dorsally on fore and mid femora, apicoposteriorly on hind femur, as well apically on tarsomeres. Hind femur incrassate, short setose, predominantly brownish-black, except paler at base and apex, 2.5 times longer than wide (Fig 10E), preapical anteroventral flange with 7–9 very strong spiny setae, and preapical posteroventral row with 3–5 strong spiny setae; hind tibia with long, sharp anteroventral carina almost reaching the apex, and less sharp posteroventral carina, present only in the basal half, ventro-basal area between two carinae concave.

*Abdomen*. Abdomen, long (1.4 times longer than mesonotum), slender, brownish-black (or reddish-brown) (Fig 17A), with paler posterior margin of tergite 4. Tergites finely granulate, except posteriorly on tergite 1 where smooth. Hairs on tergites short, adpressed except for long silver, thick hairs anterolaterally on tergite 2, predominantly black, except for whitish hairs on tergite 1, anterolaterally on tergite 2, laterally on tergites 3–4, and on pruinose fasciae on tergites 2–4. Whitish microtrichia present on tergite 1, and on tergites 2–4 as wide oblique fasciae, not reaching lateral margins (except on tergite 3 where almost touching lateral margins): V shaped on tergite 2 and with concave anterior margin on tergites 3–4. Tergite 4 large (almost as long as wide), with lateral sides protruded and of golden lustre. Sterna with greyish microtrichia and scarce, short whitish hairs, except on sternite 4 where hairs longer and yellowish. Sterna 2–3 narrow (about 1/3 width of segment); sternite 4 with semicircular incurvation posteriorly, as long as 1/4 of the length of sternite (Fig 13N); hairs yellowish, posterolateral corners covered with a tuft of yellow hairs directed from lateral sides to the centre.

*Male terminalia:* Anterior surstylar lobe with finger like projection (Fig 16C:ap) between twisted microtrichose apical (Fig 16C:aa) and innerly setose posterior part (Fig 16C:ai); posterior surstylar lobe almost covered with strong hairs on inner side, in a shape of wide rectangle with rounded protruded setose end towards cercus (Fig 16C:c); interior accessory lobe of posterior surstylar lobe velvet like (covered in fine microtrichia), consist of larger elongated part that lies on subepandrial sclerite (Fig 16C:ie) and small upper lobe (Fig 16C:iu) that on ventral margin has strong hairs; cercus in a shape of quarter circle (Fig 16C:c). Hypandrium with three small humps on wide base (Fig 16D), both anteriorly and posteriorly, and large lateral short setose areas (Fig 16D:lb); hamus sickle shaped (Fig 16D:h) with apex covered in longer microtrichia; ejaculatory apodeme large, fan-like (Fig 16D:ea).

**FEMALE.** Similar to the male except for normal sexual dimorphism (Figs 17B, 18B) and for the following characteristics: frons wide (0.28 width of head) (Fig 7D), densely granulose, shiny black except for whitish pruinose vitta along eye margins gradually becoming wider from level of antenna towards ocellar triangle, covered with scarce, thin whitish hairs; vertex without microtrichia in front of anterior ocellus; postpedicel larger, ca. 1.4 times longer than wide and 1.7 times longer than pedicel (Fig 5E); postalar callus without brown setae, only with whitish hairs; abdomen paler, can be reddish-brown, with pale posterior margin of tergite 5; sternite 4 predominantly covered with black, short, adpressed hairs.

Length: body 7–10 mm, wing 5.5–6.5 mm.

***Material examined.*** *Holotype:* **Namibia**, Katima Mulilo District, Salambala forest at 17°50’04.0“S, 24°36’13.5”E, 18–20.xi.2012, 926m a.s.l., leg. A. H. Kirk-Spriggs (Malaise traps Miombo & Mopane woodlands), (1♂: BMSA(D) 45024) (Entomology Dept. National Museum P. O. Box 266, Bloemfontein 9300) (BMSA), (DNA 110C04 K. Jordaens RMCA 2014), (det. K. Jordaens & M. de Meyer as *Eumerus* sp.).

*Paratypes*: Namibia, Katima Mulilo District, Salambala forest at 17°50’04.0“S 24°36’13.5”E, 926 m a.s.l., 18–20.xi.2012, leg. A. H. Kirk-Spriggs (Malaise traps Miombo & Mopane woodlands), (1♀: BMSA(D) 45023), (Entomology Dept. National Museum P. O. Box 266, Bloemfontein 9300) (BMSA), (DNA 110C05 K. Jordaens RMCA 2014), (det. K. Jordaens & M. de Meyer as *Eumerus* sp.); Namibia, Okahandja District, 26–27.xi.1996, leg. Irwin et al. (1♂: DNA voucher specimen Lab. code MZH_S428, G. Ståhls) (CSCA); **Botswana**, SE222680 Farmers Brigade, ca. 8 km SE of Serowe, 1300 m a.s.l., *Acacia tortilis* woodland, forestry nursery, Malaise trap, 31.viii.84, leg. P. Forchhammer, det. Lyneborg 2006 as an unpublished paratype of *Eumerus forchhammeri* (1♂: NMSA-DIP 53495) (NMSA); Botswana, 40 miles SW Ghanzi, 19.xi.1961, leg. Haacke, det. Lyneborg 2006 as an unpublished paratype of *Eumerus forchhammeri*, (1♀: NMSA-DIP 41655) (NMSA).

***Type locality:*** Namibia.

***Distribution:*** Namibia and Botswana (Fig 1).

***Biology:*** Flight period: August and November. Habitat: Miombo & Mopane woodlands, *Vachellia* (syn. *Acacia*) *tortilis* (Forssk.) Galasso & Banfi woodland, mosaic natural vegetation. Phytochoria: Kalahari-Highveld regional transition zone, KarooNamib regional centre of endemism, Zambezian regional centre of endemism.

***Etymology:*** The name “***setifemoratus***”, as an arbitrary combination, is derived from Latin nouns *seta* meaning brush and *femur* – the third segment of leg, which refers to the long and dense hairs of the fore femur.

#### *Eumerus tessellatus* Hull, 1964.

(Figs 4F, 5F, 6D, 7E, 8F, 9F, 10F, 11E, 12F, 13O, 16E,F, 17C,D, 18C,D)

***Diagnosis.*** Species with reddish-brown or brownish-black abdomen (Fig 17C,D) and light-brown/orange antenna. Ridge on the median surface of pedicel about the same distance from dorsal and ventral margin; consequently upper bare silver microtrichose area and lower setose area that is divided by the ridge are almost the same size (Figs 4F, 5F). Fore legs with short hairs (Figs 8F, 9F). Cross vein sc-r present. Katepisternum predominantly covered with setose granulae, not only on dorsoposterior corner, but distributed also deeply towards anterior and ventral margins (Fig 12F). Tergites reddish-brown to various extent, with three pairs of oblique pruinose fasciae on tergites 2–4. Male terminalia: posterior surstylar lobe in a form of long stairs, densely setose on the inner side, but less so on anterior angle (Fig 16E:pl); cercus in a shape of upright rectangle (Fig 16E:c). Very similar species to *Eumerus lacertosus* from which can be distinguished by entirely short setae on fore legs (Figs 8F, 9F), without the tuft of longer hairs on fore femur posterobasally, larger ocellar triangle with posterior ocelli located in the posterior half (Fig 6D) (in *E*. *lacertosus* ocellar triangle is small, posterior ocelli very close to each other and located in anterior half of the vertex (Fig 6B)), and by the male terminalia features. From other species with short setose fore legs, female can be discerned by the following characters: from *E. argentipedicellus* sp. nov. by the absence of any long hairs on fore femur basally and from *E. brunnipennis* sp. nov. by translucent wing. DNA barcode sequences deposited under accession numbers: XXX-XXX (S1 Table).

***Redescription.*** MALE. *Head.* Eyes contiguous for the length of 10 facets, bare (Fig 6D). Face dark brownish-black with scattered granulae, covered with dense silver microtrichia and whitish hairs (pruinescence scattered only in lower part laterally, along oral margin within small trapezoid area); contour slightly concave in lateral view, with oral margin slightly raised. Frons completely covered with dense, silver microtrichia like velvet film, and whitish hairs only along eye margin. Vertex narrow (0.2 width of head), black, shiny, with scattered granulation; covered with predominantly black hairs; whitish pruinescence present anteriorly, and a small macula posterolaterally behind posterior ocelli; plus dorsally on occiput small indistinct ellipsoidal macula along posterior eye margin behind eye corner. Ocellar triangle narrow, isosceles (distance between posterior ocelli 2 times smaller than between anterior and posterior ocelli). Occiput narrow, black-bluish, shiny dorsally, and with silver pruinescence laterally; covered with light yellow hairs dorsally (missing narrowly along eye corner) and whitish hairs laterally. Antenna light-brown/orange; arista dark brown; postpedicel rhombus-like, with narrow outer fossette along anterior margin, orange-brownish with dark dorsal margin and apex ventrally pointed, 1.3 times longer than wide and 1.7 times longer than pedicel; pedicel on inner side square like, ridge on the median inner surface of pedicel at the same distance from dorsal and ventral margins, consequently upper bare silver microtrichose area and lower setose area that are divided by the ridge are almost the same size (Fig 4F).

*Thorax.* Mesonotum blackish (Fig 17C), except for lighter posterior half of postpronotum and postalar callus brown; densely, distinctly granulate (especially scutellum), covered with short adpressed predominantly blackish hairs with bronze lustre, except anteriorly and anterolaterally where whitish; strong setae at wing basis and postalar callus black; notopleural suture present; pruinescence indistinct but present on: anterior half of postpronotum, anterior margin of scutum from postpronotum to submedial vitta, at the transverse suture as two maculae (one indistinct behind the meeting point with notopleural suture and another distinct at the end of transverse suture), and additionally three longitudinal vittae (one medial and two submedial vittae, ending at the posterior 4/5 of scutum) plus two short oblique vittae at the level of postalar calli (in some specimens submedial and oblique vittae can lack), and on anterior margin of scutellum; scutellum with about 30 granulae on posterior margin. Pleura dark brownish-black, predominantly covered with white-greyish microtrichia and on the following parts with distinct granulae and whitish hairs: anepisternum, anepimeron, proepimeron, katepisternum and metasternum. Setose granulae on katepisternum widely distributed, also present anteriorly and ventrally from hairs on the dorsoposterior corner, deeply towards anterior and ventral margin of sclerite. Wing translucent, with yellowish pterostigma, densely microtrichose, except on the following areas with reduced microtrichia: cell bc, basal parts of basal cells r and bm, and bare area near to anterobasal corner of alula; veins brown, except basal parts where yellowish; cross-vein sc-r present. Halteres yellowish. Legs predominantly dark brownish-black, except orange-yellowish on following parts: apex of fore and mid coxae; trochanters; apex of femora; basal half and apex of fore and mid tibiae, base and apex of hind tibia; fore and mid tarsi (apical two tarsomeres can be darker), plus ventral surface of hind tarsus. Hairs on legs short (Fig 8F) (except for longer hairs on mid femur posterodorsally), predominantly whitish, except anteriorly on fore and mid trochanters, and dorsally and anteriorly on fore and mid femora, basodorsally and apicodorsally on hind femur, apicodorsally on hind tibia, as well apically on tarsomeres where they are black. Hind femur incrassate, predominantly brownish-black with golden lustrous anteriorly, 2.3 times longer than wide (Fig 10F), preapical anteroventral flange with 9–10 very strong spiny setae, and preapical posteroventral row with 3–4 strong spiny setae; hind tibia with long, sharp anteroventral carina almost reaching the apex (Fig 10F), and less sharp posteroventral carina present only in the basal half.

*Abdomen.* Abdomen long (1.5 times longer than mesonotum), slender (Fig 17C). Tergites reddish-brown or brownish-black, finely granulate. In specimens with reddish-brown abdomen: tergite 1 bicoloured, in anterior half dark brownish-black, granulate and with short whitish hairs; while orange-red in posterior half and with smooth surface; tergites 2 and 3 orange-red (tergite 3 can be orange-brown). Tergite 4 brownish-black with paler area posteromedially, large (as long as wide, or slightly longer), with lateral sides ventrally protruded and of golden lustrous. Hairs on tergites short, adpressed except for long silver, thick hairs anterolaterally on tergite 2; predominantly black, except on tergite 1, anterolaterally on tergite 2, laterally on tergite 4, and on pruinose bands on tergites 2–4 where whitish. Whitish pruinescence present on tergite 1, and on tergites 2–4 as wide fasciae, not reaching lateral margins: V shaped on tergite 2, oblique on tergites 3–4, with concave anterior margin. Sterna 2–3 narrow (ca. 1/3 width of segment), with greyish pruinescence and scarce, short whitish hairs, except on sternite 4 where longer and yellowish. Sternum 4 with V shaped incurvation posteriorly (0.4 of the length of sternite); hairs yellowish, directed from lateral sides to the centre; posterolateral corners covered with tuft of yellow hairs (Fig 13O).

*Male terminalia:* Anterior surstylar lobe complex, consists of inner setose lobus (Fig 16E:ai) and apical twisted, microtrichose structure (Fig 16E:aa) with curved spine in between (Fig 16E:ap); posterior surstylar lobe rectangle in a form of longer stairs (Fig 16E:pl), densely setose innerly, but less so on anterior angle; interior accessory lobe of posterior surstylar lobe consists of microtrichose, elongated and pointed part that lies on minis (Fig 16E:ie), and small upper strongly setose part (Fig 16E:iu); cercus in a shape of upright rectangle (Fig 16E:c). Hypandrium with lateral, setose areas (Fig 16F:lb) and small upper bulge medially, and three bulges anteriorly (central is the biggest) (Fig 16F:cb) on wide basal part; microtrichose near the place where apical end of aedeagus emerge; hamus sickle-shaped (Fig 16F:h) with microtrichose apex (microtrichia minute); ejaculatory apodeme very large, fen like (Fig 16F:ea).

***Description.* FEMALE.** *Head*. Eye bare; frons wide (0.27 width of head) (Fig 7E), finely granulate and whitish setose, shiny black except for whitish pruinose vitta along eye margins; vertex without pruinescence in front of anterior ocellus. Face dark brownish-black with scattered granulae, covered with dense silver microtrichia and whitish hairs (pruinescence scattered only in lower part laterally, along oral margin within small trapezoid area); contour slightly concave in lateral view, with oral margin slightly raised. Ocellar triangle longer than wide, distance between posterior ocellus and eye margin is the same as the distance between posterior ocelli. Occiput narrow, black, shiny dorsally, and with silver pruinescence laterally. Antenna light-brown/orange; arista dark brown; postpedicel large, almost as long as wide and 2 times longer than pedicel. pedicel on inner side square like, ridge on the median inner surface of pedicel at the same distance from dorsal and ventral margins, consequently upper bare silver microtrichose area and lower setose area that are divided by the ridge are almost the same size (Fig 5F).

*Thorax.* Mesonotum black (Fig 17D), except lighter on posterior half of postpronotum and postalar callus brownish; densely, distinctly granulate, covered with short adpressed predominantly blackish hairs. Pruinescence indistinct, with similar pattern as in male. Pleura dark black, predominantly covered with white-greyish microtrichia and on the following parts with distinct granulae and whitish hairs: anepisternum, anepimeron, proepimeron, katepisternum and metasternum. Wing translucent, with yellowish pterostigma, densely microtrichose, except on the following areas with reduced microtrichia: cell bc, basal parts of basal cells r and bm, and bare area near to anterobasal corner of alula; veins brown, except basal parts where yellowish; cross-vein sc-r present. Halteres yellowish. Leg coloration and hairs as in male but with barely visible black hairs on the indicated parts. Hind femur incrassate (Fig 11E), predominantly brownish-black with golden lustrous anteriorly, 2.3 times longer than wide, preapical anteroventral flange with 9–10 very strong spiny setae, and preapical posteroventral row with 3–4 strong spiny setae; hind tibia with long, sharp anteroventral carina almost reaching the apex (Fig 11E), and less sharp posteroventral carina present only in the basal half.

*Abdomen.* Abdomen elongated (Fig 17D). Tergites reddish-brown or brownish-black, finely granulate. Coloration, hairs and pruinose fasciae of tergites 1–3 as in male; tergite 3 short, about two times wider than long; tergite 4 predominantly blackish with narrowly reddish anterior margin, with short adpressed black hairs, whitish laterally and on pruinose bands. Sternites 2–4 narrow, with greyish pruinescence and scarce, short whitish hairs; sternite 4 predominantly covered with black, short, adpressed hairs, as also sternite 5.

Length: body 10–11 mm, wing 6–7 mm.

*Variability.* Some specimens are overall darker with darker antenna (only apical half of pedicel or anterior margin of pedicel and basal square area of postpedicel orange, the rest dark brown), darker legs (all tibiae mainly dark brown, except apex and base; all tarsi brown dorsally, especially of hind leg) and dark, brownish-black tergites.

***Material examined.*** *Holotype:* RSA, Western Cape, Cape Province, Cape Peninsula, Hout Bay Skoorsteenkop, 2.ii.1951, No. 166, (Insect trap), (Swedish South Africa Expedition, 1950–1951, leg. Brinck & Rudebeck), (det. F. M. Hull *Eumerus tessellatus* Hull) (Zool. Mus. Lund Sweden, Syrphidae, type no. 3020:1), (ZML. 397 2004) (1♂: MZLU 096 2019), (Photo 2019 by MZLU) (MZLU).

*Additional material:* RSA, Eastern Cape, Cape Province, 15km SE Kirkwood, 3325DA, open bushveld shrub, 4.xi.1978, leg. R. Miller & J. Londt (1♂: NMSA-DIP 67191) (det. Lyneborg 2006 as an unpublished paratype of *Eumerus granulatus*); RSA, Eastern Cape, Cape Province, Clifton Farm 22km NW Grahamstown, 3326AB, arid area, 3.&5.i.1986, leg. J. & B. Londt and D. Gess (1♀: NMSA-DIP 67186) (det. Lyneborg 2006 as an unpublished paratype of *Eumerus granulatus*) (NMSA); RSA, Eastern Cape, Graaff-Reinet, Urquhart Caravan Park, -32.237778, 24.528333, 760 m a.s.l., succulent rocky slopes, 26–28.x.2004, leg. J. & A. Londt, (1♂: NMSA-DIP 65121), (DNA 111E05 K. Jordaens RMCA 2014) (NMSA), RSA, Eastern Cape, Willowmore, 15. xii. 1915 (1♀: NMSA-DIP 59738, det. Doesburg as *E*. *lunatus* ♀), 15. viii. 1920 (1♂: NMSA-DIP/ 49916, det. Doesburg as *E*. *lunatus* ♂), 28. xii. 1920 (1♀: NMSA-DIP 48819, det. Doesburg as *E*. *lunatus* ♀) 25. iii. 1920 (2♂: NMSA-DIP 59735, NMSA-DIP 49915), 15. viii. 1920 (1♂: NMSA-DIP 59732), 23.ix.1920 (3♂: NMSA-DIP 59734, NMSA-DIP 59733, NMSA-DIP 49937), 5.x.1920 (1♂: NMSA-DIP 49903), 20.xi.1920 (1♂: NMSA-DIP 49928), Zuurberg Range, north of Addo, 10.iv.1961 (1♂: NMSA-DIP 55819), legs. B. & P. Stuckenberg (NMSA), Albany distr., 7.ii.–21.iii.1928 (5♂, NMSA-DIP 52651, NMSA-DIP 52643, NMSA-DIP 52654, NMSA-DIP 81807, NMSA-DIP 52455, det. Doesburg as *E*. *lunatus* ♂, 5♀: NMSA-DIP 52653, NMSA-DIP 52656, NMSA-DIP 52655, NMSA-DIP 52657, NMSA-DIP 59737, det. Doesburg as *E*. *lunatus* ♀), leg. A. Walton (NMSA), Somerset, 25.ix.1925 (1♀: NMSA-DIP 48536), leg. Brauns (NMSA); RSA, Western Cape, Stellenbosch, 25.ix.1920 (1♀: NMSA-DIP 51523), (1♀: NMSA-DIP 59736, det. Doesburg as *E*. *lunatus* ♀) leg. Brauns (NMSA); Oudtshoorn District, Moeras Rivier Farm (209), 33°48’S 22°03’E, 525 m a.s.l., early September 2007, dry Karoo scrub with flowers, leg. G. Davies (1♂: NMSA-DIP 75215/DNA 111E02 K. Jordaens RMCA 2014, 1♂: NMSA-DIP 75212/DNA 114F01 K. Jordaens RMCA 2014, 1♂: NMSA-DIP 75213/DNA 111F01 K. Jordaens RMCA 2014) (NMSA); Western Cape, Gamkaskloof (Die Hel) at -33.363472, 21.627500, 336m a.s.l., Malaise traps Karoo and valley *Acacia* woodlands, 16–18.x.2012, leg. A. H. Kirk-Spriggs (1♀: BMSA(D) 39842), (Entomology Dept. National Museum P. O. Box 266, Bloemfontein 9300), (DNA 110B01 K. Jordaens RMCA 2014), (det. K. Jordaens & M. de Meyer as *Eumerus* sp.) (BMSA), Mpumalanga, Piet Retief, leg. Brauns (1♀: NMSA-DIP 48875, det. Doesburg as *E*. *lunatus* ♀).

**Type locality:** South Africa, Western Cape.

**Distribution:** South Africa (Western Cape, Eastern Cape, Mpumalanga) (Fig 1).

**Biology:** Flight period: September-October, January-February. Habitat: coastal fynbos, dry Karoo succulent and *Vachellia* (syn. *Acacia*) woodlands. Phytochoria: Kalahari-Highveld regional transition zone, Tongaland Pondoland regional mosaic, Cape regional centre of endemism, Karoo Namib regional centre of endemism.

**Note:** Species has been known only by the single male with brownish-black abdomen and brownish antenna. Lyneborg distinguished taxon with red-brown tergites as a separate new species *Eumerus granulatus* Lyneborg, but never published. Analysis of the male terminalia shows that *E. granulatus* is conspecific with *E. tessellatus*. Further more, study of the material from RSA deposited at NMSA determined as *E. lunatus* by Doesburg proved to be *E. tessellatus.* Type locality of *E. lunatus* (Fabricius, 1794) is Barbaria (NW Africa) that belongs to Palaearctic region, consequently *E. lunatus* should not be considered as Afro-tropical species. The only known type of *E. lunatus* is deposited in ZMUC, but in very bad condition (according to photo that has been provided by curator Thomas Pape only part of the thorax and one wing remain; katepisternum is not extensively granulated like in members of *triangularis* group). We suppose that Stackelberg (1961) erroneously included characters of *E. tessellatus* under name *E. lunatus* in the key, and stated that the range of *E. lunatus* is Africa instead of North Africa.

#### *Eumerus triangularis* Hervé-Bazin, 1913.

(Figs 4G, 5G, 8G, 9G, 10G, 11F, 12G, 13P, 16G,H,I, 17E,F, 18E,F)

***Diagnosis.*** Black species (Figs 17E,F, 18E,F) with three pairs of oblique pruinose fasciae on tergites 2–4. Antenna black: pedicel elongated (usually two times longer than wide and almost as long as postpedicel), on outer side anterior margin of pedicel straight in male; a setose lower area and silvery-white pruinose upper area of about the same size; postpedicel trapezoidal, with ventrally protruded apex, ended almost under right angle (in female slightly larger and with more rounded corners) (Figs 4G, 5G). Fore legs with short hairs, except moderately long hairs on fore femur posterobasally (Figs 8G, 9G). Cross vein sc-r present. Katepisternum predominantly covered with setose granulae, not only on dorsoposterior corner, but distributed also deeply towards anterior and ventral margins (Fig 12G). Sternum 4 at the posterior end with a pair of lateral, elongate, curved, finger like projections covered with long dark brownish-black hairs that surround genital opening (Fig 13P). Male terminalia: posterior surstylar lobe slightly wavy and striated in the middle of outer side, with rounded setose protrusion towards cercus (Fig 16G,H:pl), covered posteriorly on inner side with strong hairs; cercus semicircular with additional medial finger like lobus (Fig 16G,H:pp). DNA barcode sequences deposited under accession numbers: XXX-XXX (S1 Table).

***Redescription.*** *Head.* Hairs whitish (yellowish dorsally). Eye bare, in male contiguous for the length of 8–9 facets. Face contour concave in lateral view, with oral margin raised. Frons silver pruinose like velvet film, and with yellowish hairs only along eye margin (in female frons shiny, only narrowly pruinose near eye margin, covered with granulae and thin white-yellowish hairs, 0.3 width of the head). Vertex mostly shiny, except pruinose on anterior end and with pruinose lateral macula near the eye margin behind posterior ocellus continuing into lateral marginal vitta (in some specimens vitta lack). Ocellar triangle isosceles. Antenna black; postpedicel trapezoidal, with moderately wide outer fossette along anterior margin and ventrally protruded apex, ended almost under right angle, ca. 1.35 longer than wide and 1.2 times longer then pedicel (in female slightly larger and with more rounded corners, 1.25 times longer than wide, 1.8 times longer than pedicel); pedicel elongated (in female slightly shorter), usually two times longer than wide, on outer side anterior margin of pedicel straight in male, but curved in female, longitudinal ridge separates a setose lower area from silvery-white pruinose upper area of about same size (Fig 4G). Occiput moderately wide medio-dorsally and narrow laterally, black shiny dorsally, and with silver pruinescence along eye margin (distinct pruinose macula along posterior eye margin near the dorsal eye corner), except at dorsal eye corner where shiny. *Thorax*. Mesonotum black (Fig 17E), densely granulate, covered with short adpressed predominantly yellowish hairs, except on transversal fascia where mostly black at the wing level, and in front of scutellum where they are black; strong setae at wing basis and postalar callus black; notopleural suture present; pruinescence indistinct but present on: postpronotum, anterior margin of scutum from postpronotum to submedial vitta, along notopleural suture, at the transverse suture as two maculae (Fig 17E) (one indistinct behind the meeting point with notopleural suture and another distinct at the end of transverse suture), and additionally three longitudinal vittae (one medial and two submedial vittae, ending at the posterior 4/5 of scutum) plus two short lateral oblique vittae at the level of postalar calli and on anterior margin of scutellum; scutellum with about 20–25 granulae on posterior margin. Setose granulae on katepisternum widely distributed, also present anteriorly and ventrally from hairs on the dorsoposterior corner, deeply towards anterior and ventral margin of sclerite (Fig 12G). Wing brownish, with yellowish pterostigma and darker area below cross-vein sc-r, densely microtrichose; veins brown, except basal parts where yellowish; cross-vein sc-r present. Halteres yellowish. Legs dark brownish-black, except orange-yellowish at joints and ventral surface of tarsi. Hairs on legs short (except for moderately long hairs at base of fore femur posteroventrally and longer hairs on mid femur posterodorsally), predominantly whitish. Hind femur incrassate, 2 times longer than wide, preapical anteroventral flange with 10–12 very strong spiny setae, and posteroventrally with two remarkably long and strong spiny setae; scattered, moderately long hairs present ventrally; hind tibia with two long carinae almost reaching the apex (sharp anteroventral and less sharp posteroventral). *Abdomen*. Abdomen black, long, slender, with whitish pruinescence on tergite 1, and wide oblique pruinose fasciae on tergites 2–4, not reaching lateral margins (V shaped on tergite 2) (Fig 17E). Hairs on tergites short and adpressed, except for long silver, thick hairs anterolaterally on tergite 2; predominantly black, except whitish on tergite 1, on microtrichose bands and laterally on tergites 2–4 (plus posteromedially on tergite 4). Sterna narrow, with greyish pruinescence and with scarce, short whitish hairs, except for dark brownish-black long hairs on the conspicuous posterolateral, curved, finger-like projections that surround the genital opening on sternite 4 (Fig 13P). *Male terminalia:* Anterior surstylar lobe very complex, consists of inner pointed setose lobus (Fig 16G,H:ai) and apical distinctly twisted, microtrichose structure (Fig 16G,H:aa) with finger like protrusion in between (Fig 16G,H:ap); posterior surstylar lobe slightly wavy and wrinkled in the middle of outer side, with rounded setose protrusion towards cercus (Fig 16G,H:pl), covered posteriorly on inner side with strong hairs; interior accessory lobe of posterior surstylar lobe consists of microtrichose, elongated (Fig 16G,H:ie) and small upper setose part (Fig 16G,H:iu); cercus semicircular with additional medial finger like lobus (Fig 16G,H:pp). Hypandrium microtrichose near the place where apical end of aedeagus emerge; basally with small lateral setose bulges (Fig 16I:lb) and one big bulge anteriorly in the centre (Fig 16I:cb); hamus sickle-shaped (Fig 16I:h) with microtrichose apex (microtrichia minute); ejaculatory apodeme large, fen like (Fig 16I:ea).

Length: body 7–10 mm, wing 5–7 mm.

*Variability.* Pruinescence of vertex posteriorly varies, from only maculae present behind posterior ocelli to well developed lateral marginal vittae (present in the holotype and allotype and some additional specimens). Length of pedicel varies from 1.6–2 times longer than wide.

***Material examined.*** *Holotype:* **DR Congo**, Bukama, 18.iv.1911, leg. Dr. Bequaert (Musée du Congo), (1♂: R. DÉT. D 67), (*Eumerus triangularis* Hervé-Bazin *♂* cotype) (KMMA). *Allotype*: Élisabethville (Lubumbashi), 5.iv.1912, leg. Dr. Bequaert (Musée du Congo), (1♀: R. DÉT. D 67), (*Eumerus triangularis* Hervé-Bazin *♀* type) (KMMA).

*Additional material:* RSA, Free State, Rhodes, Zastron, -30.277887, 27.223028, 1.xii.2017, leg. A. Vujić, S. Radenković & N. Veličković (3♂♂: ZA4_036, ZA4_037, ZA4_038) (FSUNS); RSA, Free State, Malutizicht Lodge near Ficksburg, 8–21.iii.2011, 28°44’25.0“S, 28°03’30.0”E, 1620m a.s.l., montane grassland & bush, leg. J. G. H. & A. Londt (2♀♀: NMSA-DIP 75051/DNA 1071A04 K. Jordaens RMCA 2016; NMSA-DIP 75044/DNA 1071A05 K. Jordaens RMCA 2016); RSA, Western Cape, Ceres area, Bain’s Kloof pass, 9.xii.2017, -33.601023, 19.108318, leg. A. Vujić, S. Radenković & N. Veličković (2♂♂: ZA4_007, ZA4_009) (FSUNS); RSA, Western Cape, Ceres area, Wittebrug Nature Reserve, 12.xii.2017, -33.418071, 19.294718, leg. A. Vujić, S. Radenković & N. Veličković (2♂♂: ZA4_010, ZA4_012) (FSUNS); RSA, KwaZulu-Natal, Coleford Nature Reserve, 17.x.2015, -29.956957, 29.47227, leg. Vujić et al. (2♀♀: ZA1_140, ZA1_144 (FSUNS); KwaZulu-Natal, near Underberg, Coleford Nature Reserve, near offices, -29.956639, 29.471750, 1490m a.s.l., 17.x.2015, leg. X. Mengual (1♀: ZFMK DIP 00012299) (ZFMK); RSA, KwaZulu-Natal, Otto’s Bluff road, -29.500777, 30.36381, leg. Vujić et al. (1♀: ZA1_302, FSUNS); RSA, KwaZulu-Natal, Drakensberg mountain, Cathedral peak, blue pools, -28.945861, 29.200750, 7.xii.2012, leg. G. Ståhls, (1♀: 05773/ EU_369, http://id.luomus.fi/GJ.6396) (MZH); KwaZulu-Natal, Ladysmith Hunter’s Lodge garden, -28.554722, 29.771639, 1025m a.s.l., 26.ix.2015, leg. X. Mengual (1♂: ZFMK DIP 00012222) (ZFMK); RSA, Mpumalanga, Barberton, Fortuna trail, -25.794341, 31.049374, 19.x.2016, leg. A. Vujić (1♂: 13347) (FSUNS); RSA, Mpumalanga, Barberton, near Noordkaap, -25.667553, 31.076783, 600m a.s.l., 4.iv.2018, leg. A. Vujić, J. Ačanski, M. Đan & B. Lothrop (1♂: ZA5_237) (FSUNS); RSA, Mpumalanga, Barberton area, forest road right to Agnes Myn, small valley, -25.837000, 30.947583, 1325m a.s.l., 7.x.2015, leg. X. Mengual (1♀: ZFMK DIP 00012245) (ZFMK); RSA, Mpumalanga, Waterval Boven, near Elands River, -25.6345, 30.326065, 1400m a.s.l., 6.iv.2018, leg. A. Vujić, J. Ačanski, M. Đan & B. Lothrop (4♂♂: ZA5_245, ZA5_246, ZA5_247, ZA5_251) (FSUNS); RSA, Mpumalanga, Barberton near Aloe Ridge Guest Farm, -25.737222, 30.978056, 27.xi.2017, leg. G. Ståhls & E. Rättel (4♂♂: http://id.luomus.fi/GJ.6397, http://id.luomus.fi/GJ.6398, http://id.luomus.fi/GJ.6399, http://id.luomus.fi/GJ.6400) (MZH).

**Type locality:** Congo.

**Distribution:** Congo, RSA (Mpumalanga, Free State, KwaZulu-Natal, Western Cape) (Fig 1).

**Biology:** Flight period: March/April, September-December. Habitat: Fynbos and montane grassland. Phytochoria: Cape regional centre of endemism, Mediterranean/Sahara regional transition zone, Tongaland Pondoland regional mosaic, Zambezian regional centre of endemism.

#### *Eumerus villeneuvei* Hervé-Bazin, 1913.

(Figs 4H, 5H, 6E, 7F, 8H, 9H, 10H, 11G, 12H, 13R, 16J,K, 17G,H, 18G,H)

***Diagnosis.*** Black species (Figs 17G,H, 18G,H) with three pairs of wide oblique microtrichose fasciae on tergites 2–4. Antenna brownish-black; pedicel in male almost as long as postpedicel, with longitudinal ridge placed closer to ventral than to dorsal margin, thus silvery-white pruinose, upper area consequently much larger than setose lower area (in female ridge on pedicel separates equally sized areas or even ventrally setose area is slightly bigger); postpedicel rhomboid-shaped with ventrally protruded apex (in female larger than pedicel contrary to male) (Figs 4H, 5H). Fore legs with short setae, except with moderately long hairs at base of fore femur posteroventrally gradually becoming shorter towards apex (Figs 8H, 9H). Cross vein sc-r present. Katepisternum largely covered with setose granulae, extending from dorsoposterior corner towards ventral margin (Fig 12H). The only species of the group with extra long hairs on sternites arranged in two longitudinal, lateral rows: long whitish hairs semi-erect on sternites 2 and 3, and erect on sternite 4, especially striking in male (hairs on sternite 4 in female short, mixed whitish and black). Sternum 4 in male with a narrow V-shaped incurvation and broadly rounded posterolateral lobes that are covered with long, whitish hairs (Fig 13R). Male terminalia: Anterior surstylar lobe complex, consists of inner almost triangular strongly setose lobus (Fig 16J:ai) and apical twisted, microtrichose structure (apically finger like) (Fig 16J:aa) with spiny protrusion in between (Fig 16J:ap); posterior surstylar tapering with rounded apex (Fig 16J:pl), covered in strong hairs only near the ventral margin on inner side; cercus oval (Fig 16J:c). Hypandrium with basal part very wide, circle shaped (Fig 16K).

***Redescription.*** MALE. *Head.* Hairs predominantly whitish (yellowish dorsally). Eye bare (Fig 6E), in male contiguous for the length of 10–11 facets. Face silver-white pruinose and setose, covered with long whitish hairs that extend oral margin, contour almost straight in lateral view. Frons silver pruinose like velvet film, without hairs (in female frons densely granulate, 0.25 width of the head, shiny medially and with wide lateral pruinose vitta along eye margin). Vertex narrow (0.16 times width of head) with almost parallel lateral sides, black, densely granulate especially anteriorly, covered with whitish hairs (a few black hairs can be present on ocellar triangle), pruinose at anterior end and behind posterior ocelli till posterior margin, except for a small bare shiny area in the centre (vertex in female without anterior microtrichia but with lateral pruinescence, the most developed just behind posterior ocelli); ocellar triangle isosceles, situated in anterior half. Antenna brownish-black; pedicel in male almost as long as postpedicel, with longitudinal ridge placed closer to ventral than to dorsal margin, thus silvery-white pruinose, upper area consequently much larger than setose lower area (in female ridge on pedicel separates equally sized areas or even ventrally setose area is slightly larger); postpedicel of rhomboid shape with ventrally protruded apex and moderately wide outer fossette along anterior margin, 1.2 longer than wide (in female slightly larger and with more rounded corners, 1.25 times longer than wide and ca. 1.6 times longer than pedicel) (Fig 4H). Occiput moderately wide medio-dorsally and narrow laterally, black shiny dorsally, and with silver pruinescence along eye margin (conspicuous pruinose macula along posterior eye margin near the dorsal eye corner), except at dorsal eye corner where shiny. *Thorax*. Mesonotum black (Fig 17G), densely granulate, covered with short adpressed predominantly yellowish hairs, a few black hairs can be present at the wing level and in front of scutellum; strong setae at wing basis black (whitish on postalar callus); notopleural suture present; pruinescence distinct, present on: postpronotum, anterior margin of scutum from postpronotum to submedial vitta, laterally until wing basis, at the beginning of transverse suture and macula at the end of transverse suture, and additionally three longitudinal vittae (one medial and two submedial vittae, ending at the posterior 4/5 of scutum) plus two intra-alar vittae starting from transverse suture and ending as large semicircular spot in front of postalar callus, and on posterior end as two small triangular areas in front of scutellum; scutellum with about 25–30 granulae on posterior margin. Katepisternum covered with setose granulae from dorsoposterior corner to the ventral margin (Fig 12H). Wing translucent with slightly brownish tinge, yellowish pterostigma, microtrichose, except on the following areas with reduced microtrichia: cell bc, basal half of basal cells r above and below vena spuria, basal end of cells c, bm and cup, and anteriorly on alula; veins brown, except basal parts yellowish; cross-vein sc-r present. Halteres yellowish with brown macula dorsally on capitulum. Legs light brownish-orange, except for black femora (paler base and apex), sub-apical dark ring on mid and hind tibiae (fore tibia all light brownish-orange, without dark ring) and apical two tarsomeres darker. Hairs on legs predominantly short (except with moderately long hairs at base of fore femur posteroventrally gradually becoming shorter towards apex, longer hairs on mid femur posterodorsally and long hairs ventrally on hind femur (Fig 8H)), predominantly whitish, except for short black hairs dorsally and anteriorly on fore and mid femora, basodorsally and apically on hind femur, as well with a few black hairs apically on tarsomeres. Hind femur incrassate, 2.2 times longer than wide, with a long whitish hairs ventrally (Fig 10H); pre-apical anteroventral flange with 8–9 strong spiny setae, and posteroventral row with 2–3 strong spiny setae hidden in the hairs. Hind tibia with black sharp anteroventral carina reaching the basal half and less sharp posteroventral carina a little bit shorter. *Abdomen*. Abdomen black, long, 1.7 times longer than mesonotum, abruptly narrowing from tergite 3 (tergite 4, 1.25 times longer than wide) (Fig 17G). Tergites granulate, with whitish microtrichia on tergite 1, and wide oblique pruinose fasciae on tergites 2–4, reaching lateral margins on tergites 2 and 3 (V shaped on tergite 2, with concave anterior margin on tergite 3, the most oblique and tapering towards posterior end on tergite 4). Hairs on tergites short and adpressed, except for long silver, thick hairs anterolaterally on tergite 2; predominantly black, except whitish on tergite 1, on microtrichose bands and laterally on tergites 2–4, and a few posteromedially on tergite 4). Sterna with long whitish hairs laterally, semi-erect on sternites 2 and 3, and erect on sternite 4 forming two lateral longitudinal rows (hairs on sternite 4 in female shorter, mixed whitish and black). Sternum 4 in male with a narrow V-shaped incurvation and broadly rounded posterolateral lobes that are covered with long, whitish hairs (Fig 13R). *Male terminalia:* Anterior surstylar lobe complex, consists of inner almost triangular strongly setose lobus (Fig 16J:ai) and apical twisted, microtrichose structure (apically finger like) (Fig 16J:aa) with spiny protrusion in between; (Fig 16J:ap) posterior surstylar tapering with rounded apex (Fig 16J:pl), covered in strong hairs only near the ventral margin on inner side; interior accessory lobe of posterior surstylar lobe consists of elongated and curved part that lies on minis (with very long microtrichia along margin and apically) (Fig 16J:ie), and upper small rounded strongly setose part (hairs scattered and long) (Fig 16J:iu); cercus oval (Fig 16J:c). Hypandrium with rounded apex (Fig 16K), microtrichose near the place where apical end of aedeagus emerge, circle shaped at base (in dorsal view), with two lateral setose bulges (Fig 16K:lb) and one big bulge anteriorly in the centre (Fig 16K:cb); hamus sickle-shaped (Fig 16K:h) with microtrichose apex (microtrichia minute); ejaculatory apodeme large, fen like (Fig 16K:ea).

Length: body 9–10 mm, wing 5–6 mm.

***Material examined.*** *Holotype:* **DR Congo**, Katanga, Katwamba (type locality situated at 8°S–28°E according to Hervé-Bazin (1913: 69)), 10.xi.1911, leg. Dr. Bequaert, Musée du Congo (1♂: R. DÉT. C 67) (KMMA).

*Additional material:* **Malawi**, Kasungu National Park, Lifupa Camp 1333Aa, 1000m a.s.l, *Brachystegia* (Fabaceae) tree, 9–10.xii.1980, leg. B. Stuckenberg & J. G. H. Londt, det. Lyneborg 2006 as *Eumerus villeneuvei* H.-B. (1♂: NMSA-DIP 67124) (NMSA); **Botswana**, Serowe, Malaise trap, 16.vi.1983, leg. P. Forchhammer, det. Lyneborg 2006 as *E. villeneuvei* H.-B. (1♀: NMSA-DIP 31299) (NMSA); **Tanzania**, prov. Mbeya, 80km NW of Tunduma, -8.822, 32.285, 1385m a.s.l., 6.xii.2017, leg. J. Halada (1♂: RU300/22757) (M. B. coll); Tanzania, prov. Rukwa, 10 km SE of Mpui, -8.405, 31.798, 1750m a.s.l., 4.xii.2017, leg. J. Halada (1♀: RU301/22691) (MBPC); Tanzania, prov. Kigoma, 15km NE of Nyaka Kangaga, -4.081, 30.549, 1180m a.s.l., 29–30.xi.2017, leg. J. Halada (1♀: RU302/22758) (MBPC).

**Type locality:** Congo

**Distribution:** DR Congo, Tanzania, Malawi and Botswana (Fig 1).

**Biology:** Flight period: June, November/December. Habitat: Miombo woodlands, *Brachystegia* Benth. tree. Phytochoria: Zambezian regional centre of endemism, Kalahari-Highveld regional transition zone, Lake Victoria regional mosaic.

### Identification key for species of *Eumerus triangularis* group

1. Katepisternum with extra granulae and hairs also present anteriorly and/or ventrally from the setose dorsoposterior corner (as on Fig 12). Pedicel internally enlarged, squarish or elongated, usually divided into two triangular areas: upper bare, silver-white pruinose area and lower setose area (as on Figs 4, 5) (except in *Eumerus brunnipennis* sp. nov. with dorsal pruinose area also almost all covered in hairs, Figs 4B, 5B) *................* ***Eumerus triangularis* group.**
  - Katepisternum with hairs restricted to dorsoposterior corner and area near ventral margin, or rarely completely covered in granulae. Pedicel without above cited pattern *.................................................................................................* **other Afro-tropical species of genus *Eumerus.***

***E. triangularis* group.**

1. Eyes holoptic (male) *.....................................................................................................................* **2.**
  - Eyes dichoptic (female) *........................................................................................................* **10.**
2. Inner surface of pedicel almost entirely covered with strong, short hairs, including upper silver-white pruinose area (except for area near dorsal margin where bare or with scattered hairs) (Fig 4B). Black species with strong brownish coloration of wing (Fig 2C). Fore legs with short hairs (Fig 8B). Setose granulae on katepisternum mainly present on dorsoposterior corner and ventrally, and just few in between and towards anterior end. Male terminalia (Fig 13C,D): cercus enlarged, with additional short setose bulge (Fig 13C:pb); posterior surstylar lobe stairs like, striated medially (Fig 13C:pl), posteroventrally densely setose on inner side *...............................................................................................* ***E. brunnipennis* sp. nov.**
  - Inner surface of pedicel divided into upper silver-white pruinose triangular area (without hairs) and lower setose area (as on Fig 4A,C–H) *.....................................................................* **3.**
3. Fore trochanter ventrally and basal third of fore femur posteroventrally with long and curved whitish hairs (as on Fig 8D,E) *.......................................................................................* **4.**
  - Fore trochanter and fore femur with short hairs or fore femur at the base posteroventrally with longer but straight hairs and short ones on the rest of the leg (as on Figs 8A,C,F,G,H,15B) *............................................................................................................................* **5.**
4. Scutum short setose (shorter than the longer hairs at the beginning of transverse suture). Fore femur only in basal third with long, curved whitish hairs (Fig 8E), the rest of hairs on fore femur very short; hind femur short setose (Fig 10E). Antennae orange-yellowish; pedicel innerly with upper silver-white pruinose area and lower setose area of almost the same size (Fig 4E). Male terminalia (Fig 16C,D): posterior surstylar lobe in a shape of wide rectangle with rounded protruded setose end towards cercus (Fig 16C:pl), almost all covered in strong hairs on the inner side *.................................................................* ***E. setifemoratus* sp. nov.**
  - Scutum long setose (almost as long as longer hairs at the beginning of transverse suture). Long hairs on fore femur gradually becoming shorter from base towards apex (Fig 8D); hind femur with long hairs ventrally and posteroventrally (Fig 10D). Antennae brownish-black; pedicel innerly with upper bare silver pruinose area slightly larger than setose lower area (Fig 4D). Male terminalia (Fig 5A,B): posterior surstylar lobe large, of rhomboid shape with triangular apex (Fig 16A:pl), striated medially, almost all covered in strong hairs on inner side *........................................................................................* ***E. rubidus* Hull.**
5. Pedicel elongated (ca. 1.6–2.0 times longer than wide), silvery-white pruinose upper area of about the same size as a setose lower area (Fig 4G), on the outer side anterior margin straight. Sternum 4 at the posterior end with a pair of lateral, curved, raised finger-like projections covered with long dark brownish-black hairs that surround genital opening (Fig 13P). Male terminalia (Fig 16G–I): posterior surstylar lobe slightly wavy and striated medially (Figs 16G,H:pl), with rounded setose protrusion towards cercus, covered posteriorly on the inner side with strong hairs; cercus semicircular with additional medial finger-like lobus (Figs 16G,H:pp) *.......................................................................* ***E. triangularis* Hervé-Bazin.**
  - Pedicel not elongated, almost as long as wide, or slightly longer than wide (ca. 1.2–1.3 times) (as in Figs 4A,C,F,H, 15C); on the outer side anterior margin curved. Sternum 4 without long, finger-like posterolateral projections (only lobes can be present) (as on Fig 13I,K,L,O,R). Male terminalia different *..........................................................................................* **6.**
6. Pedicel with dorsal silver-white pruinose area and ventral setose area almost of the same size (as on Figs 4F, 15C) *.....................................................................................................................* **7.**
  - Pedicel with dorsal silver-white pruinose area distinctly larger than setose ventral area (as on Fig 4A,C,H) *.....................................................................................................................................* **8.**
7. Hairs on fore femur short, without the tuft of longer hairs posterobasally (Fig 8F). Ocellar triangle larger with posterior ocelli located in posterior half (Fig 6D). Male terminalia (Fig 16E,F): posterior surstylar lobe in a form of longer stairs (Fig 16E:pl), innerly densely setose, except bare on anterior angle; cercus in a shape of upright rectangle (Fig 16E:c) (Distribution: South Africa) *..............................................................................* ***E. tessellatus* Hull.**
  - Fore femur posterobasally with a tuft of longer hairs (Fig 15B). Ocellar triangle small with posterior ocelli very close to each other, located in the anterior half of vertex (Fig 6B). Male terminalia (Fig 13G,H): posterior surstylar lobe in a form of short stairs (Fig 13G:pl), innerly densely setose including the anterior angle; cercus semicircular (Fig 13G:c) (Distribution: Yemen and UAE) *.....................................................................................* ***E. lacertosus* Smit.**
8. Sterna with very long whitish hairs laterally, semi-erect on sternites 2 and 3, and erect on sternite 4 forming two longitudinal lateral rows (stick out from lateral view). Fore legs predominantly with short setae, except for long hairs at base of fore femur posteroventrally gradually becoming shorter towards apex (Fig 8H). Male terminalia (Fig 16J,K): posterior surstylar lobe tapering with rounded apex (Fig 16J:pl), with strong hairs only near the ventral margin on inner side; cercus oval (Fig 16J:c). Hypandrium with basal part very wide, circle shaped in ventral view ............................................................. ***E. villeneuvei* Hervé-Bazin.**
  - Sterna with shorter hairs, not striking out from lateral view ..................................................... 9.
9. Mesonotum and hind femur (Fig 10A) short setose. Fore femur predominantly with short hairs, except for a few slightly longer hairs posterobasally (Fig 8A). Antenna brownish-black (Fig 4A). Male terminalia (Fig 13A,B): cercus in a shape quarter of circle (Fig 13A:c); posterior surstylar lobe in a shape of rounded rectangle (Fig 13A:pl) covered on inner side with strong hairs situated along diagonal and upper triangle; hypandrium as usual (Fig 13B) (apical part almost as long as base) ..................... ***E. argentipedicellus* sp. nov**.
  - Mesonotum and hind femur (Fig 10C) ventrally with a moderately long hairs. Fore femur with long hairs basally and moderately long hairs posteriorly (Fig 8C). Antenna orange-yellowish (Fig 4C). Male terminalia (Fig 13E,F): cercus very specific, with lateral triangular protrusions (Fig 13E:cl), all covered in long thin hairs and additionally on ventral side by long, dense, strong microtrichia; posterior surstylar lobe rhomboidal with triangular apex, covered with strong hairs only posteroventrally on inner side; hypandrium stocky (wide base, shorter apical part) (Fig 13F) ................................... ***E. clavicercus* sp. nov**.
10. Inner surface of pedicel almost covered with strong, short hairs including upper silver-white pruinose part (except for an area near dorsal margin where bare or with scattered hairs) (Fig 5B). Black species with strong brownish coloration of wing (Fig 2D). Fore legs with short setae (Fig 9B). Setose granulae on katepisternum mainly present on dorsoposterior corner and ventrally, and just few in between and towards anterior end (as in Fig 12B) ...................................................................................... ***E. brunnipennis* sp. nov**.
  - Inner surface of pedicel with upper silver-white pruinose triangular area (without hairs) and lower setose area (as on Figs 5A, C–H, 15F). Wing translucent or with brown tinge, but not with strong brownish coloration ............................................................................................. **11.**
11. Pedicel elongate, at least 1.5 times longer than wide; a setose lower area of almost the same size (or a little bit larger) as bare, silvery-white pruinose, upper area (Fig 5G). Frons with narrow white pruinose vitta along eye margin. Hairs on mesonotum short (Fig 18F). Base of fore femur with longer hairs posteroventrally (Fig 9G) and hind femur with scattered long hairs alternating short ones ventrally (Fig 11F). Tergites black (Fig 17F) ......................................................................................................................... ***E. triangularis* Hervé-Bazin.**
  - Pedicel as long as wide ..................................................................................................................... **12.**
12. Fore trochanter ventrally and basal 1/3 of fore femur posteroventrally with long and curved hairs; long hairs on fore femur gradually becoming shortly from base towards apex (Fig 9D). Katepisternum besides setose granulae on dorsoposterior corner and ventral end also with an isolated group of 6–8 setae near the centre of sclerite, which are difficult to discern (as on Fig 12D). Upper bare silver microtrichose area of inner side of pedicel almost of equal size as lower setose area (Fig 5D). Pruinose vitta along eye margin on frons widen medially (Fig 7C). Scutum covered with longer hairs, almost as long as those at the start of transverse suture. Hind femur with long hairs ventrally and even longer posteroventrally (Fig 11D). Tergites reddish-brown (Fig 2H) ....................................................... ***E. rubidus* Hull.**
  - Fore trochanter without long hairs, if fore femur with longer hairs basally than these are straight, not curved (as on Figs 9A,C,E,F,H, 15E). Katepisternum without isolated group of setae near the centre of sclerite ........................................................................................................ **13.**
13. Sterna 2 and 3 long setose. Frons with wide white pruinose vitta along eye margin (Fig 7F). Mesonotum short setose. Long hairs present posteroventrally at the base of fore femur (Fig 9H) and ventrally on hind femur (Fig 11G). Katepisternum largely granulate (as on Fig 12H). Tergites black (Fig 17H) .................................... ***E. villeneuvei* Hervé-Bazin.**
  - Sterna 2 and 3 short setose .............................................................................................................. **14.**
14. Mesonotum covered in longer hairs, almost as long as longer hairs at the beginning of transverse suture. Fore femur with longer hairs posterobasally gradually tapering towards apex (Fig 9C). Hind femur with moderately long, dense hairs ventrally (Fig 11B). Katepisternum largely granulate (as on Fig 12C). Tergites 2–4 blackish, tergite 5 paler ..................................................................................................................................................... ***E. clavicercus* sp. nov**.
  - Mesonotum covered in short hairs, much shorter than hairs at the beginning of transverse suture. ................................................................................................................................................... **15**
15. Pedicel with upper bare silver microtrichose area slightly larger than lower setose area (Fig 5A). Fore femur with only few (1–3) longer hairs posterobasally (Fig 9A). Frons with narrow pruinose vitta along eye margin. Katepisternum largely granulate (as on Fig 12A). Tergites 2–3 reddish, other tergites blackish (Fig 2B) ................. ***E. argentipedicellus* sp. nov**.
  - Pedicel with upper bare silver microtrichose area and lower setose area of almost the same size (as on Figs 5C,E, 15F) ............................................................................................................... **16.**
16. Fore femur short setose, without long hairs posterobasally (Fig 9F). Frons with narrow white pruinose vitta along eye margin (Fig 7E). Mesonotum and hind femur short setose. Katepisternum largely granulate (as on Fig 12F). Tergites 2–3 reddish, other tergites blackish (Fig 17D) ......................................................................................................... ***E. tessellatus* Hull.**
  - Fore femur posterobasally with longer hairs (as on Figs 9E, 15E) .......................................... **17.**
17. Antenna orange-yellow (Fig 5E). setose granulae on katepisternum mainly present on dorsoposterior corner and ventrally, and just few towards anterior and ventral end (as on Fig 12E) ........................................................................................................ ***E. setifemoratus* sp. nov**.
  - Antenna brownish (Fig 15F). Katepisternum largely granulate (as on Fig 15G) ........................................................................................................................................................... ***E. lacertosus* Smit**

### Molecular data analyses

The analysis of DNA barcode sequences for specimens which material was genetically examined, supported six species within the *E. triangularis* species group. The pairwise distances based on DNA barcode sequences varied from 0.058 between *E. rubidus* and *E. setifemoratus,* sp. nov. to 0.924 between *E. argentipedicellus* and *E. setifemoratus* sp. nov. (Table 1).

**Table 1:** Mean pairwise uncorrected p-distances based on the COI DNA barcode between six *Eumerus* species of the *triangularis* group (below diagonal) and intraspecific overall mean uncorrected p-distances.

	1	2	3	4	5	6	Intraspecific overall mean p-distance
1. *E. argentipedicellus* sp. nov.	/						0.010
2. *E. triangularis*	0.070	/					0.010
3. *E. tessellatus*	0.069	0.076	/				0.000
4. *E. brunnipennis* sp. nov.	0.086	0.073	0.071	/			/
5. *E. rubidus*	0.091	0.086	0.090	0.092	/		0.000
6. *E. setifemoratus* sp. nov.	0.092	0.081	0.084	0.079	0.058	/	0.000

**Fig 19:**
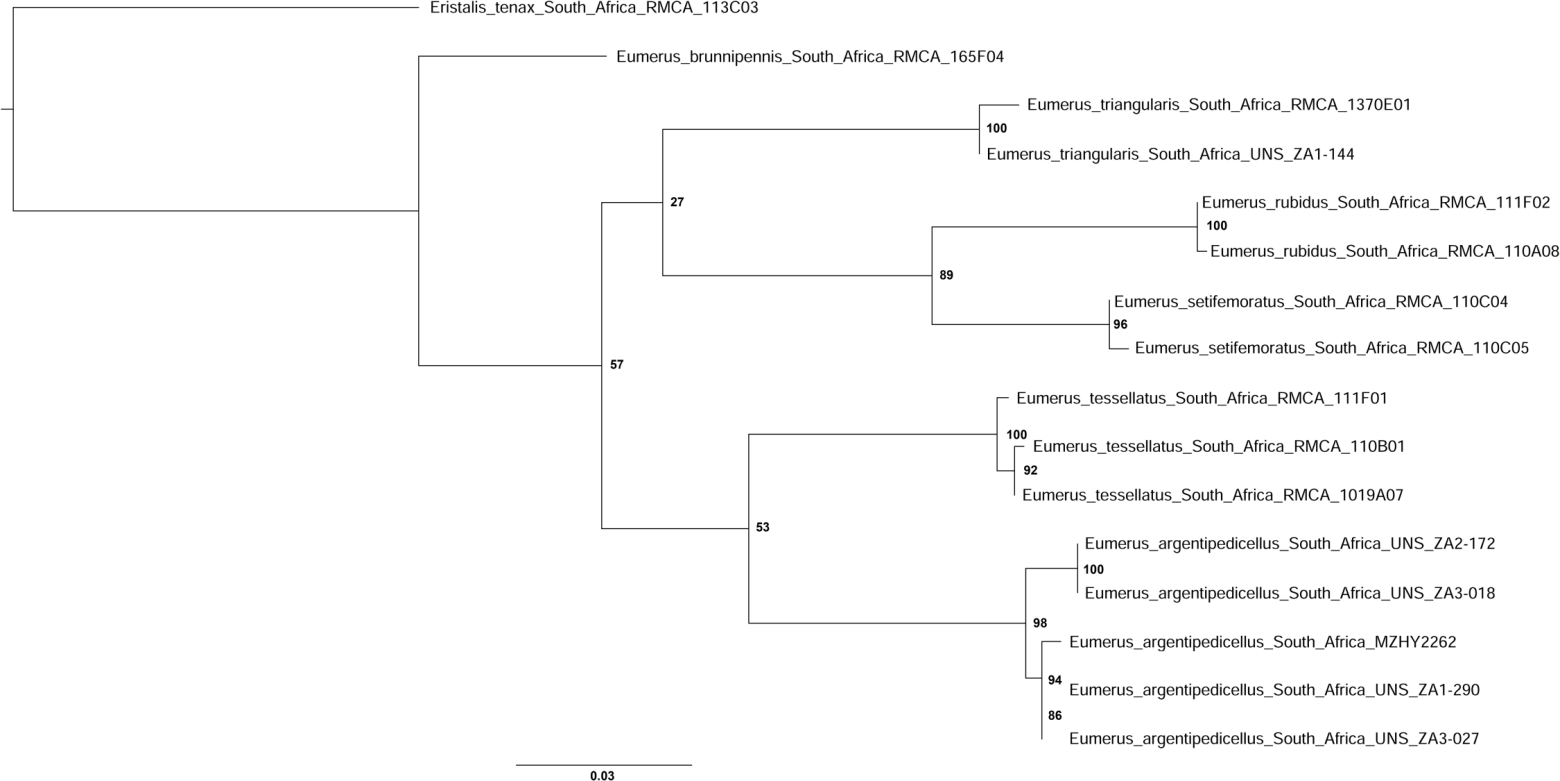
Maximum-likelihood tree based on DNA barcode (5’-end COI gene) sequences.

**Fig 20:**
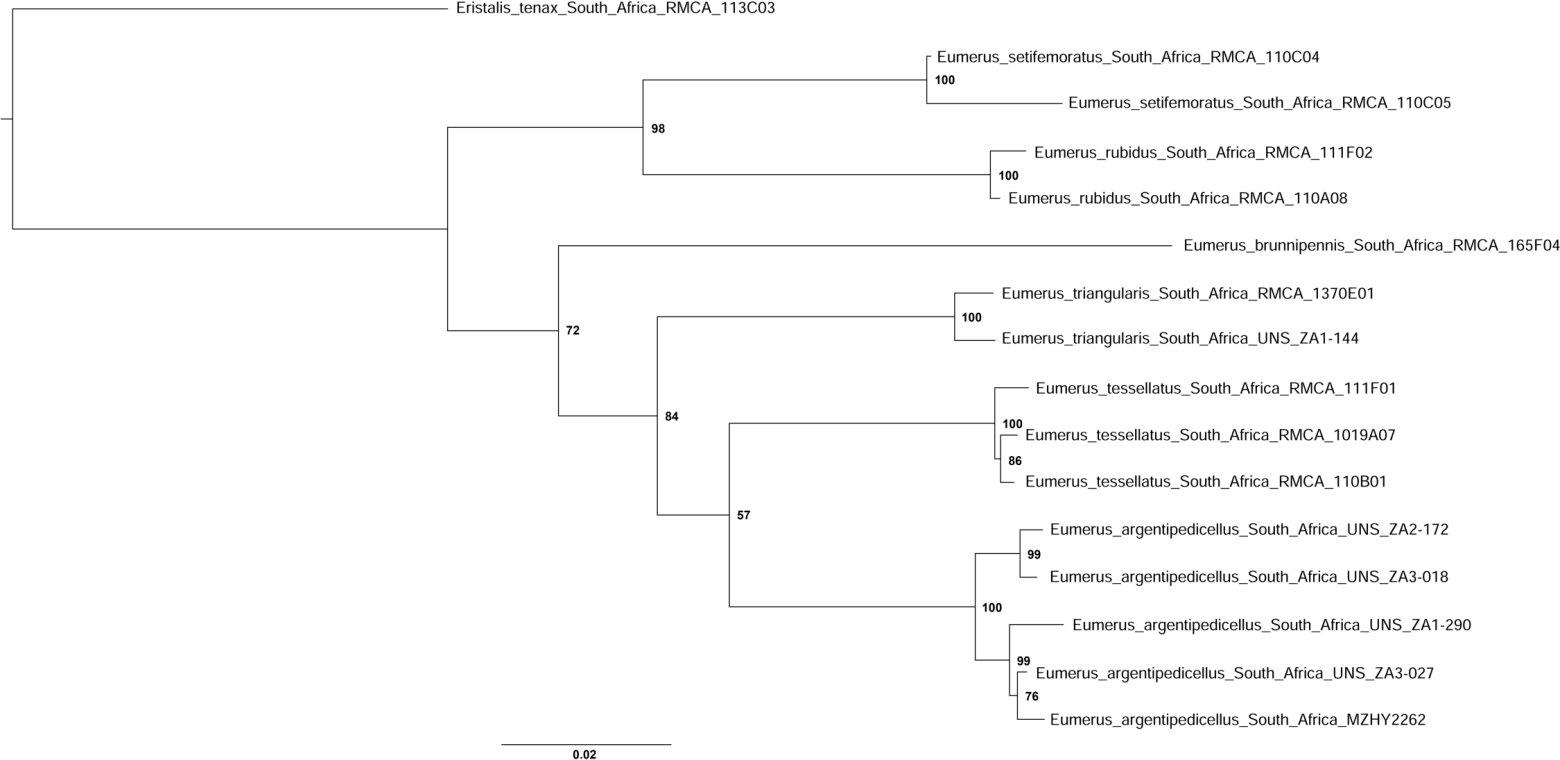
Maximum-likelihood tree based on concatenated dataset including fragments of COI, CytB and 28S rRNA genes.

The ML tree based on the 5’-end COI gene (DNA barcode region) is given in Fig 19 and shows that species form well separated clusters. The ML tree based on all molecular markers is given in Fig 20 and shows two clades. The first clade consists of species having long and curved hairs on the fore legs, *viz. E. rubidus* and *E. setifemoratus* sp. nov. The second clade comprises four species with short or moderately long and straight hairs, viz. *E. brunnipennis* sp. nov., *E. triangularis*, *E. argentipedicellus* sp. nov., and *E. tessellatus.* We could not obtain molecular data for *E. lacertosus*, *E. villeneuvi*, and *E. clavicercus* sp. nov.

## Discussion

In their study of hoverflies in the Arabian Peninsula, Smit et al. [24] classified together newly discovered Arabian species *E*. *lacertosus* with African *E*. *triangularis* and *E*. *lunatus*, as members of the same species group. They identified several diagnostic characters for this species group, including bare eye, contiguity of eye shorter than the ocellar triangle, broadly triangular postpedicel, coarsely punctuated and short-haired thorax, strongly swollen hind femur with a spinose ridge and a few large black spines apico-ventrally, very acutely pointed abdomen, and shallowly serrate margin of the scutellum. We have discovered more members of this species group, each of them from Africa, and named it *triangularis* group. The diagnosis for the species group given by Smit et al. [24] is expanded with the most striking character such as silvery-pruinose triangular upper part of the pedicel that make antenna glistering (more distinct in male) and most probably has a role in recognition between mates. Also some other features like extensively granulated katepisternum and specific male terminalia with complex structure of sustylar lobes proved to be diagnostic for this species group. Based on all available data on *E. lunatus* (original description, study of remain parts of the holotype, wrong identifications), we consider that this species from coastal North of Africa does not belong to the *E. triangularis* group.

*Eumerus triangularis* group is restricted to the Afrotropical Region (but absent from Madagascar), with the highest diversity in RSA. Only one species of the *triangularis* group, *viz. E. lacertosus*, is present out of Africa, and occurs in the south of the Arabian Peninsula. Two species of the group (*E. triangularis* and *E. villeneuvei*) are widely distributed in central and south Africa, while the other species are restricted to particular regions of RSA or, as *E. setifemoratus* sp. nov., only found in Namibia and Botswana. All species of the group are clearly delimited by morphological characters of adults and supported as independent evolutionary units by molecular data. Morphological and molecular analyses revealed two clades within the *E. triangularis* group. One clade comprised species characterised by very long, curved hairs on the fore legs, and male terminalia with the posterior surstylar lobe completely covered in hairs and with a straight ventral margin. It includes *E. rubidus* and *E. setifemoratus* sp. nov., which are primarily distributed in south-western Africa. The second clade shows a slightly different grouping when considering either morphological or molecular data. Based on morphology, it consists of two branches: i) species with completely short setose fore legs (*E. brunnipennis* sp. nov., *E. lacertosus*, *E. tessellatus*) and ii) species with moderately long, but straight hairs basally (*E. argentipedicellus* sp. nov., *E. clavicercus* sp. nov., *E. triangularis* and *E. villeneuvei*). Both of these branches possess posterior surstylar lobe partially covered in hairs and curved ventral margin. Furthermore, *E. brunnipennis* sp. nov. stands out within the group due to the presence of strong, short hairs on the entire inner surface of the pedicel (which are absent in all other species of the *triangularis* group on the upper silver-white pruinose area). Additionally, specific enlarged cercus of male terminalia distinguishes males of this species from males of all other species. This species also shows the largest interspecific p-distances of all species of the group and represents the first split in its clade. The *E. argentipedicellus* sp. nov. and *E. tessellatus* appear as sister species based on molecular data, which is also evident in similar shape of male terminalia.

The *E. triangularis* species group is predominantly distributed in southern part of the African continent, with its highest diversity in RSA, particularly along mountain ranges (Cape fold mountain system in the Western and Eastern Cape and the south-eastern highlands) is most probably connected with the extraordinary plant-species richness and high degree of floral endemism of this region. Ricarte et al. [50] showed that *Eumerus* larvae feed and live in both dicots and monocots, and in a variety of families including Orobanchaceae, Cactaceae, Euphorbiaceae and Asteraceae. They seem to prefer underground storage organs of plants of the families Xanthorrhoeaceae and Hyacinthaceae [50]. Unfortunately, immature stages of species of the *E. triangularis* group are still unknown. There are several genera of Hyacinthaceae which have their highest diversity in Southern Africa and that show a similar distribution in the Afrotropical Region as the species of the *E. triangularis* group, but rarely reach the Arabian Peninsula. One such genus is *Albuca* L., mainly distributed in southern and eastern Africa, with only a few species extending into the northern regions of Chad and Nigeria, Ethiopia and Saudi Arabia [51]. Interesting is that *E. tessellatus* distributed from the very end of the African continent is according to morphology the most similar to *E. lacertosus* restricted to the Arabian Peninsula (unfortunately for this species molecular data are missing). Further research on the host plants is necessary to reveal the life cycles of these species and the putative coevolutionary processes which underpin the rich diversity of *E. triangularis* species group in southern Africa.

## Conclusion

We address the taxonomy of the Afrotropical endemic *Eumerus triangularis* species group through an integrative approach combining morphological and molecular data. We described four new species (*E. argentipedicellus* sp. nov., *E. brunnipennis* sp. nov., *E. clavicercus* sp. nov. and *E. setifemoratus* sp. nov.), redescribe four ill-defined species in the group, and provide the first descriptions of the male of *E. rubidus* and of the female of *E. tessellatus*. Morphological and molecular results support the existence of two clades within the *E. triangularis* species group. More research with a larger molecular data set, and research on the species’ host plants is needed to get insight into the evolutionary history of this Afrotropical endemic species group.

## Supporting information

S1 Table List of molecularly analysed samples with GenBank accession numbers. (XLSX)
